# Intention to leave, depersonalisation and job satisfaction in physicians and nurses: a cross-sectional study in Europe

**DOI:** 10.1038/s41598-024-52887-7

**Published:** 2024-01-28

**Authors:** L. Maniscalco, M. Enea, N. de Vries, W. Mazzucco, A. Boone, O. Lavreysen, K. Baranski, S. Miceli, A. Savatteri, S. Fruscione, M. Kowalska, P. de Winter, S. Szemik, L. Godderis, D. Matranga

**Affiliations:** 1 Department of Health Promotion, Mother and Child Care, Internal Medicine and Medical Specialties, University of Palermo, Palermo, Italy; 2 Department of Internal Medicine, Spaarne Gasthuis, Hoofddorp, The Netherlands; 3 Spaarne Gasthuis Academy, Hoofddorp, The Netherlands; 4 Department of Public Health and Primary Care, Centre for Environment and Health, KU Leuven (University of Leuven), Leuven, Belgium; 5 Department of Epidemiology, Medical University of Silesia, Katowice, Poland; 6 Department of Psychology, Educational Science and Human Movement, University of Palermo, Palermo, Italy; 7 Leuven Child and Health Institute, KU Leuven, Leuven, Belgium; 8 Department of Development and Regeneration, KU Leuven, Leuven, Belgium; 9 Department of Pediatrics, Spaarne Gasthuis, Haarlem and Hoofddorp, The Netherlands

**Keywords:** Health policy, Occupational health, Public health, Epidemiology

## Abstract

The European healthcare sector faces a significant shortage of healthcare workers. Assessing the prevalence of this issue and understanding its direct and indirect determinants are essential for formulating effective recruitment programs and enhancing job retention strategies for physicians and nurses. A multicentric cross-sectional study was conducted, involving 381 physicians and 1351 nurses recruited from eight European hospitals in Belgium, the Netherlands, Italy, and Poland. The study focused on assessing turnover intentions among healthcare workers based on the Job Demands-Resources model, using an online questionnaire. Structural equation models were employed to test the data collection questionnaires’ construct validity and internal consistency. The turnover intention was assessed by agreement with the intention to leave either the hospital or the profession. Among physicians, 17% expressed an intention to leave the hospital, while 9% intended to leave the profession. For nurses, the figures were 8.9% and 13.6%, respectively. The internal consistency of the questionnaires exceeded 0.90 for both categories of health workers. Depersonalization and job dissatisfaction were identified as direct determinants of turnover intention, with work engagement being particularly relevant for nurses. We found a higher intention to leave the hospital among physicians, while nurses were more prone to leave their profession. To mitigate turnover intentions, it is recommended to focus on improving job satisfaction, work engagement and fostering a positive working climate, thereby addressing depersonalisation and promoting job retention.

## Introduction

The global shortage of healthcare workers (HCWs) has emerged as a critical concern with wide-ranging implications for healthcare systems worldwide. This shortage is attributed to factors such as an aging population, the increasing prevalence of patients with chronic diseases requiring continuous care, and the growing demand for healthcare services^1^. The Global Burden of Disease Study 2019 estimated a worldwide deficit of 6.4 million physicians and 30.6 million nurses, based on the minimum required numbers (i.e. 20.7 physicians and 70.6 nurses per 10,000 inhabitants) necessary to achieve 80% universal health coverage^2^. In Europe, an inadequate workforce, an ageing healthcare workforce, growing challenges in training and education, and a brain drain contribute to the shortage of physicians^3^. By 2030, the estimated healthcare workforce demand in Europe will be 18.2 million, representing + 28.1% compared to 2013^4^.

The scarcity of healthcare providers has far-reaching consequences, including extended waiting times and limited patient access to healthcare services^5^. From the perspective of HCWs, this shortage results in an increased workload, heightening the risk of errors and clinical hazards that compromise patient care^6,7^. Moreover, it carries a significant risk of chronic stress and burnout, contributing to HCWs’ turnover intentions^8^.

Turnover among nurses is highly influenced by factors such as job dissatisfaction, stress and burnout^9^. Additionally, personal and professional indicators, organisation profile, work environment, and patient-related factors have been found to significantly influence nurses’ intention to stay^10^. Regarding physicians, socio-demographic personal and family characteristics, working time, psychosocial working conditions, job-related well-being and career-related aspects are variables associated with intentions to leave^11^.

The findings of a systematic review on factors impacting nurse and physician retention in hospitals highlighted job dissatisfaction, lack of career development and work-life balance, as the main determinants of intention to leave^12^.

Identifying significant direct and indirect determinants of turnover intention can aid in the early detection of high-risk healthcare workers, enabling targeted interventions to reduce attrition and promote retention. Intention to leave has been found to be a reliable predictor of actual workforce attrition, emphasizing the importance of proactive measures to address this issue^13,14^.

To investigate turnover intention in relation to job satisfaction, work engagement, and burnout, we conducted a cross-sectional multicentre study involving nurses and physicians from eight European hospitals. The primary objective was to estimate the prevalence of intention to leave the organization and the profession. Secondly, after investigating the construct validity and internal consistency of a dedicated questionnaire developed for data collection, this study aimed to provide data-driven evidence regarding the direct and indirect determinants of intention to leave, as proposed by the adapted Job Demands-Resources (JD-R) model for turnover intention.

### Theoretical model

Within the framework of the project entitled “Mental Health: focus on Retention of healthcare workers (METEOR)”, funded by the European Health and Digital Executive Agency in 2020, we aimed to assess job satisfaction, burnout and turnover intentions, using the theoretical framework of the JD-R model^15,16^. According to this model, strain occurs when there is an imbalance between job demands and available resources to cope with those demands. Job demands encompass various factors, including physical (e.g., workload, work pace), psychological (e.g., emotional demands), social (e.g., work-life balance), and organizational (e.g., task distribution) factors. On the other hand, job resources, such as physical (e.g., sufficient staff levels), psychological (e.g., the meaningfulness of work), social (e.g., mentoring), and organizational factors (e.g., autonomy, influence at work), facilitate work goals, alleviate the burden of job demands, and promote professional and personal growth. In addition, it is known that job demands and job control levels are predicted by the core components of burnout (emotional exhaustion, depersonalization and health complaints) and by an extended engagement function^17^. In this paper we focused on the most important dimensions of burnout, exhaustion and depersonalization^18^.

**Figure 1:**
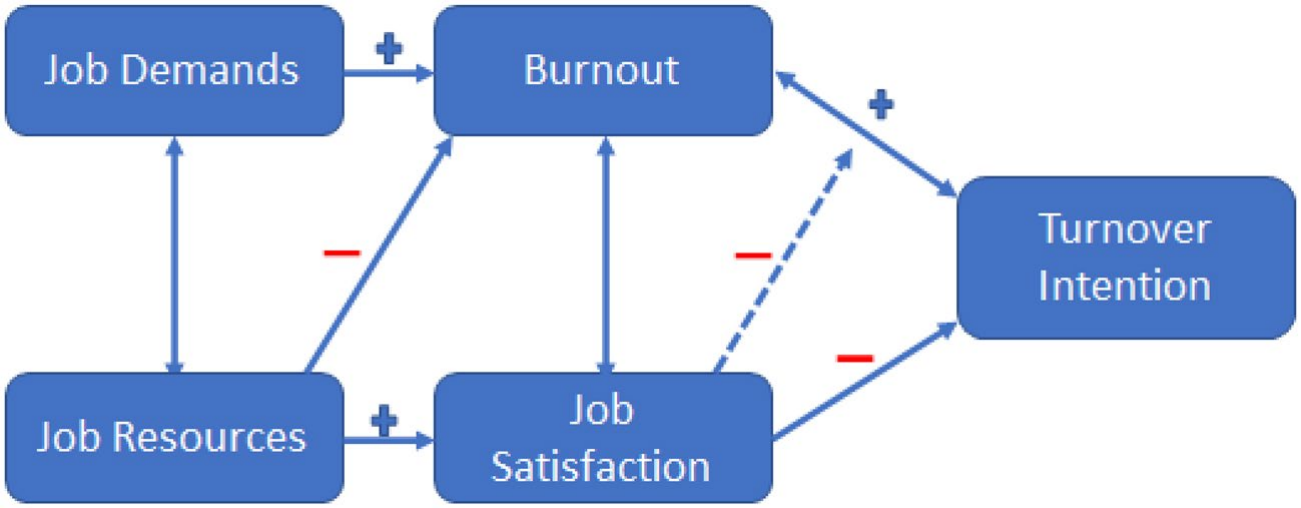
The theoretical turnover intention according to Meteor (MTI) model.

The METEOR Turnover Intention (MTI) model, which is an adaption of the JD-R Model, considers job demands and job resources influencing factors of turnover intentions, through the mediation of burnout and job satisfaction (Fig. 1). The first hypothesis posits that job satisfaction and work engagement will increase by reducing job demands, as mediated by burnout (H1). This assumption is based on the well-known result that job demands and job control are predicted by a core burnout function (positive association) and by an extended engagement function (negative association)^17,19^.

Furthermore, it has been observed that higher levels of job resources, such as increased opportunities for learning, support from co-workers, and support from supervisors^20^, are significantly associated with an increase in work engagement, particularly among nurses^21^. On this basis, the second hypothesis posits that job satisfaction and work engagement increase by increasing job resources (H2).

The third hypothesis states that burnout, specifically emotional exhaustion and depersonalisation, is a contributing factor to the intention to leave (H3). This hypothesis aligns with H1 and, hence, focuses on emotional exhaustion and depersonalization as the core components of burnout^18^. The final hypothesis asserts that job satisfaction and work engagement act as protective factors, reducing intention to leave and increasing job retention (H4). Supporting evidence for H3 and H4 are extensively described in Ref.^12^. The hypothesized model for turnover intention also assumes a positive covariance between emotional exhaustion and depersonalization^16^, as well as between job demand and resources^15^.

## Materials and methods

### Study design

Participants in this cross-sectional multicentre study were selected from two hospitals, one academic and one non-academic, in each of the following countries: Belgium, the Netherlands, Italy, and Poland. An initial invitation to partake in the study was dispatched via the intranet or email too all physicians and nurses in force at each participating hospital at the date of 1st April 2022. The immediate contact details of the participants were blinded to the research group. Fourteen days later, a reminder email was dispatched once more to all participants. In the case of the number of respondents lower than requested, the medical and nurses’ associations have been involved to solicit nurses and physicians partaking the research by explaining them the expected benefits in terms of workplace well-being. Before respondents could participate in the study, they would have to read the information letter and agree with the online informed consent. Participants had to hold a degree in Nursing (bachelor or higher) or Medicine (master) and could either work in healthcare or hold a managerial position. Other inclusion criteria were adult age and being currently working as a nurse or physician in one of the eight participating hospitals. Nurses and physicians affiliated to hospitals different than the ones included in this study and those working in EU countries different from the ones participating in this study were excluded from the study. Caring experts, midwives, psychologists, lab technicians and paramedics were excluded, too. Data were collected between May 16 and September 30, 2022. The sample size calculation aimed to estimate the proportion of individuals reporting the intention to leave their job, with a confidence level of 95% and an absolute error of 5%. Based on existing literature, the estimated proportions of intention to leave were 42.2% for nurses and 28.2% for physicians^22^. Considering a 20% non-response rate, the expected sample size was determined to be 450 nurses and 370 physicians, overall. The sample was allocated equally among the eight hospitals (56 doctors and 46 nurses per hospital).

### The questionnaire

The questionnaire was developed in English and translated into Dutch, Italian and Polish. After back translation, the questionnaire was administered through an online platform compliant with the General Data Protection Regulation (Survey Monkey). The questionnaire consisted of 75 items, with an additional question about education included only for nurses. It was structured into eight sections: individual and work environment characteristics, Job demands (JD), Job resources (JR), Work engagement (WE), Job satisfaction (JS), Emotional Exhaustion (EE), Depersonalization (DP) and intention to leave (IL). Items for JD, JR, WE and JS were derived from the Copenhagen psychosocial questionnaire (COPSOQ)^23^ and from the Hospital survey on patient safety culture (HSOPS) tool^24^. Items for EE and DP were extrapolated from the Maslach Burnout Inventory (MBI)^16^. IL was assessed through two questions. The first question aimed to gauge the intention to leave the current hospital and was formulated as “I intend to leave my current hospital for another one in the near future”. The second question targeted the intention to leave the healthcare profession and was phrased as “I intend to leave my healthcare profession for another job”. Participants were asked to indicate their level of agreement with these statements^25^.

To be consistent with the source-validated tools, items for JD, JR, WE, JS and IL were scored on a 5-point Likert scale, coded between 0 and 4 (between 4 and 0 for reversed items) while items for EE and DP were scored on a 7-point Likert scale ranging between 0 and 6. The complete questionnaire can be found in the Supplement Materials.

### Statistical methods

Each domain of JD, JR, WE, JS, EE, DP and IL, was transformed into a 0–100 scale using the sum of the component scores, divided by the maximum achievable score, and multiplied by 100^26^. Missing data were imputed using the R package “mice”, which employs multiple imputation via chained equations.

Confirmatory factor analysis (CFA) was conducted using a structural equation model with measurement component to test the MTI model. The seven domains were treated as latent variables, with two exogenous variables (JD and JR) and five endogenous variables (WE, JS, EE, DP and IL), measured by the corresponding sections of the questionnaire.

Several goodness-of-fit indices were used to select the best CFA model. Good models have RMSEA values ≤ 0.08, CFI and TLI values close to 1, SRMR close to 0 or CD close to 1. For comparison between alternative models, the difference between the chi-square values of the models was calculated, which also has a chi-square distribution. To identify significant omitted paths that could enhance the model’s goodness-of-fit, the modification index (MI) was calculated.

Internal consistency was assessed for the overall questionnaire and for each of the seven latent constructs using Cronbach’s alpha coefficient. Higher alpha values indicate greater internal consistency. Internal consistency is considered poor with alpha values below 0.60, questionable when between 0.60 and 0.70, acceptable between 0.70 and 0.80, good between 0.80 and 0.90 and excellent when higher than 0.90^27^.

All analyses were performed using R Statistical Software (v4.1.2; R Core Team 2021) and Stata IC/15.1 for Windows.

### Ethics approval

This study was performed in line with the principles of the Declaration of Helsinki. The study was approved by the Ethics Committee Research UZ/KU Leuven in January 2022 (S66009).

### Consent to participate

Informed consent was obtained from the participants, who received detailed information about the study.

## Results

Among the 543 physicians who provided informed consent for the survey, 381 (70%) completed the survey. The average age of the respondents was 44.7 (SD 10.2) years, with 229 (60%) being female (Table S1). Among the recruited physicians, 62 (16.3%) expressed their intention to leave the hospital, while 34 (8.9%) indicated their intention to leave the medical profession. Most of respondents were from Belgium (n = 158, 41%), followed by the Netherlands (n = 112, 29%), Italy (n = 105, 28%) and Poland (n = 6, 2%).

The internal consistency of the questionnaire was excellent overall (alpha = 0.939). The alpha values for the seven domains ranged from acceptable values (DEP’s alpha = 0.751, WE’s alpha = 0.732) to good values (JD, JR, JS showed alpha equal to 0.882, 0.840 and 0.844, respectively) and an excellent value for EE (alpha = 0.908). However, IL showed poor internal consistency (alpha = 0.598) (Table 1).

**Table 1:** Means, standard deviations and internal consistencies (Cronbach’s alpha) of the MTI questionnaire for physicians.

Latent construct*	Physicians (n = 381)		Nurses (n = 1351)	
	Mean (SD)	Alpha	Mean (SD)	Alpha
JD (n = 22)	57.62 (12.11)	0.882	54.93 (11.16)	0.873
JR (n = 15)	62.94 (12.73)	0.840	61.95 (11.64)	0.816
WE (n = 3)	66.89 (16.23)	0.732	67.6 (15.77)	0.787
EE (n = 9)	32.93 (20.98)	0.908	31.04 (19.9)	0.914
DP (n = 5)	13.88 (14.9)	0.751	13.12 (15.93)	0.786
JS (n = 5)	59.38 (18.94)	0.844	56.53 (16.32)	0.802
IL (n = 2)	29.27 (23.77)	0.598	29.61 (23.83)	0.725
Overall (n = 61)	48.77 (7.07)	0.939	47.06 (7.07)	0.934

*MTI* turnover intention according to *Meteor,* *JD* job demands, JR job resources, *WE* work engagement, *JS* job satisfaction, *EE* emotional exhaustion, *DP* depersonalization, *IL* intention to leave.

*All scales range between 0 and 100.

On a 0–100 scale, the respondents rated themselves in the medium range for JD (57.62 ± 12.11) and above the medium range for JR (62.94 ± 12.73), WE (66.89 ± 16.23) and JS (59.38 ± 18.94). The self-assessment for EE (32.93 ± 20.98) and IL (29.27 ± 23.77) was low, while it was very low for DP (13.88 ± 14.90) (Table 1).

**Figure 2:**
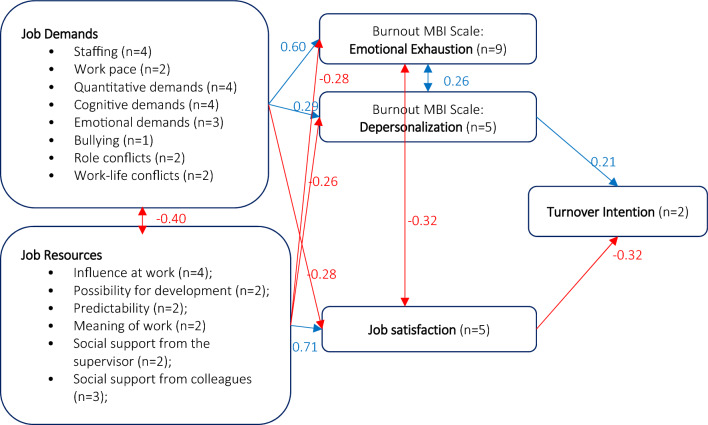
Construct validity of the MTI questionnaire in a sample of 381 physicians from eight European hospitals. In the picture there are shown the path coefficients of the latent component of the MTI SEM model for physicians. Positive effects and covariances are shown in blue, negative effects and covariances are shown in red. *MTI* Turnover Intention according to Meteor.

Based on CFA fit indices (Table S2), the best model for physicians included two direct and two indirect determinants of turnover intention (Fig. 2). The direct effects showed a positive relation between DP and turnover intention (coeff = 0.21, p-value = 0.002) and a negative relation between JS and turnover intention (coeff =  − 0.61, p-value ≤ 0.001). JD and JR had indirect effects on IL, which was mediated by DP and JS. Specifically, JD had a positive effect on DP (coeff = 0.29, p-value ≤ 0.001) and a negative effect on JS (coeff =  − 0.28, p-value ≤ 0.001). Conversely, JR had a positive effect on JS (coeff = 0.71, p-value ≤ 0.001) and a negative effect on DP (coeff ≥  − 0.26, p-value ≤ 0.001). Interestingly, WE did not have a significant direct or indirect impact on IL. In addition to significant covariances between latent constructs JD and JR (cov =  − 0.40, p-value ≤ 0.001) and between EE and DP (cov = 0.26, p-value ≤ 0.001), a significant covariance was found between EE and JS (cov =  − 0.32, p-value ≤ 0.001) (Table 2). All the path coefficients of the measurement component of the MTI model for physicians were statistically significant (Table S3).

**Table 2:** Results of confirmatory factor analysis: path coefficients of the latent component of the MTI model for physicians.

Endogenous variables	Exogenous variables	Path coefficient	95% CI		P > z
Job satisfaction	Job demands	− 0.28	− 0.37	− 0.19	< 0.001
	Job resources	0.71	0.64	0.79	< 0.001
Emotional exaustion	Job demands	0.60	0.52	0.68	< 0.001
	Job resources	− 0.28	− 0.37	− 0.19	< 0.001
Depersonalization	Job demands	0.29	0.20	0.39	< 0.001
	Job resources	− 0.26	− 0.36	− 0.16	< 0.001
Intention_to_leave	Job satisfaction	− 0.61	− 0.73	− 0.49	< 0.001
	Depersonalization	0.21	0.07	0.34	0.002

Covariance between pairs of latent variables		Covariance	95% CI		P > z
(Emotional Exhaustion, Job satisfaction)		− 0.32	− 0.45	− 0.19	< 0.001
(Emotional Exhaustion, Depersonalization)		0.26	0.16	0.35	< 0.001
(Job demands, Job resources)		− 0.40	− 0.51	− 0.29	< 0.001

*MTI* turnover intention according to *Meteor.*

Of the 1680 nurses who participated in the study and responded to the survey, 1351 (80%) provided a complete response. The average age of the respondents was 43.8 (SD 11.8) years, with 1111 (82.2%) being female (Table S4). Among the enrolled nurses, 113 (8.4%) reported the intention to leave the hospital, while 184 (13.6%) indicated the intention to leave the nursing profession. Most of respondents were from Belgium (n = 957, 63%), followed by Netherlands (n = 344, 25%), Italy (n = 86, 6%) and Poland (n = 63, 5%).

The internal consistency of the questionnaire for nurses was excellent overall (alpha = 0.934). The alpha values for the seven domains ranged between acceptable values (IL’s alpha = 0.725, DP’s alpha = 0.786, WE’s alpha = 0.787), good values (JD, JR, JS showed alpha equal to 0.873, 0.816 and 0.802, respectively) and the EE’s excellent value (alpha = 0.914) (Table 1). On a 0–100 scale, the respondents rated themselves in the middle range for JD (54.93 ± 11.16) and JS (56.53 ± 16.32), in the second half for JR (61.95 ± 11.64) and WE (67.60 ± 15.77), and in the first half for EE (31.04 ± 19.90), IL (29.61 ± 23.83) and DP (13.12 ± 15.93) (Table 1).

**Figure 3:**
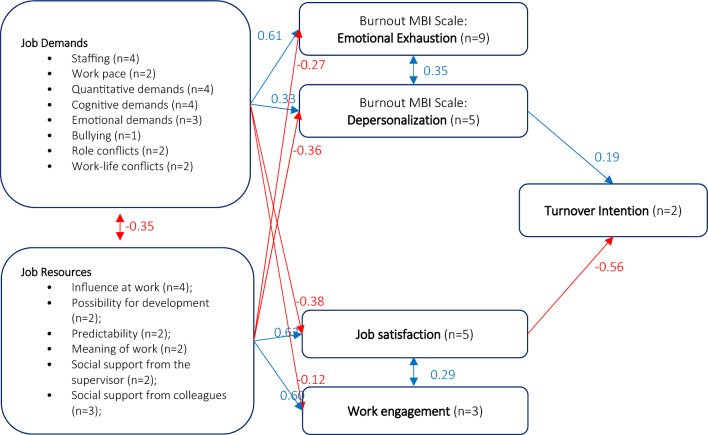
Construct validity of the MTI questionnaire in a sample of 1351 nurses from four European countries. In the picture there are shown the path coefficients of the latent component of the MTI SEM model for nurses. Positive effects and covariances are shown in blue, negative effects and covariances are shown in red. *MTI* turnover intention according to Meteor.

Based on CFA fit indices (Table S5), the best model for nurses included two direct and two indirect determinants of turnover intention (Fig. 3). The direct effects showed a positive relation between DP and turnover intention (coeff = 0.19, p-value ≤ 0.001) and a negative relation between JS and turnover intention (coeff =  − 0.56, p-value ≤ 0.001). JD and JR had indirect effects on IL, as mediated by DP and JS. Specifically, JD positively affected DP (coeff = 0.33, p-value ≤ 0.001) and negatively affected JS (coeff =  − 0.38, p-value < 0.001), while JR positively affected JS (coeff = 0.62, p-value ≤ 0.001) and negatively affected DP (coeff =  − 0.36, p-value ≤ 0.001). In addition to significant covariances between the latent constructs JD and JR (cov =  − 0.35, p-value ≤ 0.001) and between EE and DEP (cov = 0.36, p-value ≤ 0.001), significant covariance between WE and JS (cov = 0.29, p-value ≤ 0.001) were found (Table 3). All the path coefficients of the measurement component of the MTI model for nurses were statistically significant (Table S6).

**Table 3:** Results of Confirmatory factor analysis: path coefficients of the latent component of the MTI model for nurses.

Endogenous variables	Exogenous variables	Path coefficient	95% CI		P > z
Work engagement	Job demands	− 0.13	− 0.2	− 0.07	< 0.001
	Job resources	0.60	0.54	0.66	< 0.001
Job satisfaction	Job demands	− 0.38	− 0.43	− 0.33	< 0.001
	Job resources	0.62	0.57	0.67	< 0.001
Emotional exaustion	Job demands	0.61	0.57	0.65	< 0.001
	Job resources	− 0.27	− 0.31	− 0.22	< 0.001
Depersonalization	Job demands	0.33	0.27	0.39	< 0.001
	Job resources	− 0.36	− 0.42	− 0.30	< 0.001
Intention_to_leave	Job satisfaction	− 0.56	− 0.63	− 0.50	< 0.001
	Depersonalization	0.19	0.12	0.26	< 0.001

Covariance between pairs of latent variables		Covariance	95% CI		P > z
(Work engagement, job satisfaction)		0.29	0.20	0.38	< 0.001
(Emotional exhaustion, depersonalization)		0.36	0.30	0.42	< 0.001
(Job demands, job resources)		− 0.35	− 0.41	− 0.28	< 0.001

*MTI* turnover intention according to *Meteor.*

## Discussion

The objective of the study was to determine the prevalence of nurses’ and physicians’ intention to leave their respective health organizations or current professions and to identify the determinants associated with this phenomenon.

A recent systematic review^12^ reports that the prevalence of the intention to leave ranges between 11.7 and 64.4% for nurses and between 14.2 and 26.1% for physicians. A comparison of our estimates with these ranges must be interpreted in light of the definition and measurement adopted for turnover intention. In fact, the intention to leave can be related to the workplace^28^, the healthcare facility and/or the profession^29–33^, the current practice^34^, or the job itself^35,36^. Also, the timeframe used to assess the intention to leave can be very different across studies. Turnover intentions can be investigated prospectively over the next 1^37^, 2^34^, 3^28,37,38^ or 5^11^ years, or retrospectively with regards to having considered leaving a job or profession within the past year^22,39,40^. Finally, some studies do not specify a particular time frame^25,36,41^ at all. In our study we considered either the hospital or the profession as target and we adopted the near future as the time frame for the intention to leave.

Interestingly, the questionnaire’s internal consistency was proven to be overall excellent, except for the intention to leave. This inconsistency can be attributed to the fact that the intention to leave the profession and the intention to leave the hospital seem to be two different phenomena. The former suggests feelings of alienation and rejection with a negative impact on the healthcare sector (macro level), while the latter implies a desire for change that only impacts the hospital (meso level) or the ward (micro level).

Our survey found that physicians were more inclined to leave their current hospital than the medical profession, while nurses exhibited a higher intention to leave the nursing profession itself rather than their specific workplace. It could be hypothesized that among nurses, the professional practice no longer arouses a high attraction in terms of gratification and recognition of their own role concerning salary, working, or organizational conditions, especially if compared to the high emotional and physical pressures suffered in the last pandemic.

Data emerging from the literature suggested how the risk from SARS-CoV-2 in the healthcare settings was considered as an aggravated generic risk, rather than a specific risk, meaning that the workers are more exposed to SARS-CoV-2 in terms of intensity and frequency of contact with other potential infected individuals than the ones from other working settings^42,43^. In this cross-sectional study, both categories were under pressure, but the higher percentage of nurses expressing the intention to leave the profession compared to physicians suggests that nurses may have been more significantly affected by the pandemic. Previous reports have already highlighted that nurses experienced high levels of burnout, depression, anxiety, and stress during the COVID-19 period, and were more likely to consider leaving their jobs compared to physicians^44^. The main stressors for nurses included increased workload, dealing with death, stigmatization, occupational stress and exposure, the risk of infection^45^, and high levels of COVID-19-related discrimination^46,47^. This survey highlighted the turnover intention of physicians also, for whom far fewer studies were available to date^12^.

Our study aimed to examine the direct and indirect determinants influencing the intention to leave among physicians and nurses working in hospital settings as related to job satisfaction, work engagement and the burnout. The study hypotheses were confirmed only in part and varied between physicians and nurses.

Specifically, job demands were associated with increased emotional exhaustion and depersonalization, as well as with decreased job satisfaction for both physicians and nurses (H1 confirmed). Similarly, job resources were linked to decreased emotional exhaustion and depersonalization, as well as increased job satisfaction for both groups of healthcare workers (H2 confirmed).

However, the hypothesis suggesting that burnout would act as a driving force for the intention to leave (Hypothesis 3) was not supported by either physicians or nurses. In fact, only depersonalization, and not emotional exhaustion, was found to be a direct determinant of the intention to leave, along with job satisfaction.

Furthermore, the results suggested that the intention to leave among physicians was not explained by work engagement (H3 rejected). Rather, a significant portion of the variability was explained by the negative correlation between job satisfaction and emotional exhaustion. On the other hand, work engagement played a role in explaining the intention to leave among nurses, as it was positively correlated with job satisfaction (H4 confirmed).

For both workers’ categories, feeling depersonalized and reporting low job satisfaction represented two direct determinants of the intention to leave. Indirectly, job demands affected the intention to leave and job satisfaction, while job resources acted as protective factors. An increase in job demands was related to increased depersonalization, thereby reducing job satisfaction. On the contrary, increased job resources resulted in increased job satisfaction and reduced depersonalization while mitigating job strain and burnout^48^.

According to the JD-R model, work engagement is influenced by the availability of job resources, while burnout is associated with job demands more than job resources. Interestingly, the absence of a direct or indirect impact of work engagement on physicians’ intentions to leave could be attributed to the disproportionate burden of job demands. Factors such as role ambiguity, role conflict, role stress, stressful events, workload, and work pressure^49^ outweigh the available resources, including task variety, task significance, autonomy, feedback, and social support^50^.

The motivating process triggered by perceived resources determines positive outcomes such as work engagement and high organizational commitment, as they help to achieve goals, reduce job demands, and promote personal growth and development^17^. Therefore, when workers have access to numerous job resources, they can withstand high work demands.

Conversely, in the case of nurses, work engagement was found to be related to job satisfaction. This suggests that regardless of the internal or external resources available, the degree of satisfaction experienced in the workplace plays a crucial role in determining positive outcomes in terms of performance and commitment. This highlights the importance of targeted and functional interventions aimed at promoting well-being in the workplace, focusing on a particular need expressed by nurses. This finding aligns with the current literature, which shows that among nurses job satisfaction is positively associated with work engagement^51^, making it a significant determinant of turnover intention^52,53^.

In both physicians and nurses, depersonalization alone, and not emotional exhaustion, played a role in explaining the intention to leave. Emotional exhaustion, in some circumstances, is experienced as a mental state inherent in the work activity, while depersonalization appears to be more related to the sphere of psychopathology, representing a significant risk factor for the HCWs’ health^54^. The experience that occurred during the SARS-CoV-2 pandemic probably remains vivid among healthcare workers, with emotional exhaustion not undermining their commitment to continue their profession but rather reinforcing their bond to professional practice in emergency conditions.

The main strength of this study is including countries that provided a geographically balanced representation of different regions in Europe. In fact, the selected countries represent a wide range of healthcare systems: in Belgium there is a mix combining compulsory, comprehensive and universal public health insurance with freedom of choice and independent medical practice; in the Netherlands, the health demand is covered by basic insurance and long-term nursing and care, that are two statutory forms of insurance; in Poland, the mandatory health insurance is the primary source of public funding, which however is undersized in relation to the increasing population needs. Lastly, the Italian National Health Service is inspired by the principles of universality, equality and equity through the provision of essential levels of care accessible free of charge to the whole population and in a uniform way. The last available Euro Health Consumer Index score (2018) was the highest in the Netherlands (883), while Belgium (849), Italy (687), and Poland (585) showed lower scores. Compared to the EU average health expenditure in proportion to GDP (9.8%), Belgium and the Netherlands invested a higher proportion of their GDP on health (10.3% and 10.0%, respectively), while Italy (8.8%) and Poland (5.2%) ranked below the EU average.

This study provided a validated tool to assess the intention to leave phenomenon in European hospital HCWs.

One study limitation is the imbalance in the number of physicians and nurses, which may have contributed to some differences in the significant determinants of turnover intention. However, the lower response rate of physicians, as compared to nurses, is not uncommon on this topic, and previous studies have also observed this imbalance. For example, the two HCWs’ categories have been represented in a 1:3 ratio^36^, 1:4 ratio^22,55^ or 1:6 ratio^39^ in former studies. This imbalance could also be responsible for a selection bias that can occur if the most motivated subjects to partake in the survey were, at the same time, those less likely to intend to leave. Similarly to our study, a high response rate has often been found associated with a low percentage of turnover intentions^41,56,57^. This would suggest that the real extent of the phenomenon for physicians could have been underestimated by our survey. Moreover, the cross-section study design prevents us from making interpretations of causal relations among determinants of turnover intentions. Finally, further research will be addressed to investigate worker’s and hospital’s level determinants for turnover intention and to deepen differences across countries.

## Conclusions

The study provided estimates on the prevalence of the intention to leave the hospital and the profession among physicians and nurses, highlighting at the same time how the hospital context determined a greater turnover intention in doctors, while for nurses the discomfort appeared to be prevalently linked to the professional practice itself.

Furthermore, our findings suggested the important role of depersonalization in explaining the intention to leave in both the HCWs’ categories and confirmed previous studies indicating work engagement as a determinant of nurses’ intention to leave.

In conclusion, based on the evidence provided by this cross-sectional study, the management at the micro (ward), meso (hospital) and macro (policy) levels are called to define appropriate recruitment and retention policies, specifically for nurses and physicians, in consideration that job satisfaction, work engagement and a good working climate, they all appear to be strategic factors in promoting the job retention.

### Supplementary Information

Supplementary Information.

## Data Availability

The datasets used and analysed during the current study are available from the corresponding author on reasonable request.
