# Supplementary material for: Intention to leave, depersonalisation and job satisfaction in physicians and nurses: a cross-sectional study in Europe
Source: Sci Rep. 2024 Jan 28;14:2312. doi: 10.1038/s41598-024-52887-7 (PMC10822871; doi:10.1038/s41598-024-52887-7)
Supplement: Supplementary file 1 — Supplementary Information. [file 41598_2024_52887_MOESM1_ESM.docx]

Supplementary Material

Intention to leave, depersonalisation and job satisfaction in physicians and nurses: a cross-sectional study in Europe

Laura Maniscalco ^†1^, Marco Enea ^†1^, Neeltje de Vries^2,3^, Walter Mazzucco^1^, Anke Boone^4^, Olivia Lavreysen^4^, Kamil Baranski^5^, Silvana Miceli^6^, Alessandra Savatteri^1^, Santi Fruscione^1^, Malgorzata Kowalska^5^, Peter de Winter^7,8,9^, Szymon Szemik^5^, Lode Godderis^4^, and Domenica Matranga*^1^

*** Correspondence:** Domenica Matranga: [domenica.matranga@unipa.it](mailto:domenica.matranga@unipa.it)

In Sections 1-4 there are the METEOR questionnaires. In sections 5-10 there are supplementary tables

1. **English questionnaire**
   1. **Questionnaire for physicians: current work experience**

Dear Madam / Sir,

We appreciate your participation in this survey regarding the **working conditions, general health and well-being of physicians.** METEOR is a project funded by the European Union’s Health Programme (2014 - 2020) (Grant Agreement 101018310). METEOR's mission is to improve the mental health and well-being of European health workers.

We would like to ask you to respond to **a series of questions** on your perceptions of various areas of your work environment, such as your relationship with other health professionals, your workload and staffing and your intention to leave the profession (if any).

The questionnaire should be completed **within three weeks** after getting this invitation and it should take approximately **15 minutes** to complete. As a physician with firsthand knowledge of your hospital, your replies are critical and will be used to improve patient care and your work environment.

Your responses to the questions are **strictly personal and confidential.** Each question will require a response. Kindly select the option that most accurately expresses your current feelings or opinions about the subject. During the three-week survey period, you may access the survey at any time and at your convenience. You are only permitted to complete the survey once.

Kind regards,

*[Contact details of each partner according to the country where the survey is submitted]*

**ABOUT YOU**

|  | Gender | - Male - Female - Other |
| --- | --- | --- |
|  | Age | …………………….. |
|  | How would you describe your current living situation? | - Living alone - Living together with partner - Living with partner and child(ren), - Living with friends / housemates - Living with parents - Other…................. |

**Work context**

|  | Home Hospital | - UZ Leuven Academic hospital, Belgium - AZ Delta, Belgium - Medical University of Silesia, Poland - SP ZOZ Szpital Wielospecjalistyczny w Jaworznie, Poland - Amsterdam University Medical Centre, the Netherlands - Spaarne Gasthuis, the Netherlands - Palermo University Hospital, Italy - Local Health Facility of Agrigento, Italy | |
| --- | --- | --- | --- |
|  | Form of employment | - Full-time - Part-time (anything <100%) | |
|  | Type of contract | - Permanent - Non-permanent (temporary) | |
|  | How many years have you been working in this hospital? | - <=1 year - 2 years - … - >40 | |
|  | Do you do nightshifts? | - Yes - No | |
|  | Specialty area (please, consider your current job) | - Anaesthesia and resuscitation - Cardiology - Dermatology and venereology - Physical Medicine & Rehabilitation - Gastroenterology - Forensic Medicine - Geriatrics - Gynaecology and obstetrics - General Surgery - Internal Medicine - Child and adolescent psychiatry - Psychiatry - Clinical biology - Oncology - Neurosurgery - Neurology - Ophthalmology - Nuclear Medicine - Ophthalmology - Orthopaedic Surgery - Otorhinolaryngology, head and neck surgery - Pathological anatomy - Paediatrics - Plastic, reconstructive and aesthetic surgery - Pneumology - Radiotherapy - Rheumatology - Radiology - Stomatology, Oral and Maxillofacial Surgery - Emergency Medicine - Urology - Occupational Medicine - General Practice - Hygiene and preventive medicine - Other… (specify) | |
|  | Have you been involved as frontline with Covid-19 patients? | - Yes - If No, goes to the next section | |
|  | How long did you work as frontline with Covid-19 patients? | - Less or equal than 1 year - More than 1 year | |
|  |  |  |  |

**Working conditions**

|  | The hospital provides all the materials I need to do my job at best | - Strongly agree - Agree - Neither agree nor disagree - Disagree - Strongly disagree | |
| --- | --- | --- | --- |
|  |  |  |  |

**Health problems during your last job**

|  | Have you suffered from any illness, disability or other physical or mental problem caused or made worse by work, apart from the occupational injuries, in your last job in the past three years? | - Yes - No | |
| --- | --- | --- | --- |
|  |  |  |  |

**STAFFING**

|  | We have enough staff to handle the workload | - Strongly agree - Agree - Neither agree nor disagree - Disagree - Strongly disagree | |
| --- | --- | --- | --- |
|  | Staff in this unit work longer hours than is best for patient care. | - Strongly disagree - Disagree - Neither agree nor disagree - Agree - Strongly agree | |
|  | We use more temporary staff than is best for patient care. | - Strongly disagree - Disagree - Neither agree nor disagree - Agree - Strongly agree | |
|  | We work in "crisis mode" trying to do too much, too quickly. | - Strongly disagree - Disagree - Neither agree nor disagree - Agree - Strongly agree | |
|  |  |  |  |

**WORK PACE**

|  | Do you have to work very fast? | - Never - Seldom - Sometimes - Often - Always | |
| --- | --- | --- | --- |
|  | Do you work at a high pace throughout the day? | - To a very small extent - To a small extent - Somewhat - To a large extent - To a very large extent | |
|  |  |  |  |

**QUANTITATIVE DEMANDS**

|  | Is your workload unevenly distributed so it piles up? | - Never - Seldom - Sometimes - Often - Always | |
| --- | --- | --- | --- |
|  | How often do you not have time to complete all your work tasks? | - Never - Seldom - Sometimes - Often - Always | |
|  | Do you get behind with your work? | - Never - Seldom - Sometimes - Often - Always | |
|  | Do you have enough time for your work tasks? | - Always - Often - Sometimes - Seldom - Never | |
|  |  |  |  |

**COGNITIVE DEMANDS**

|  | Do you have to keep your eyes on lots of things while you work? | - Never - Seldom - Sometimes - Often - Always | |
| --- | --- | --- | --- |
|  | Does your work require that you remember at lot of things? | - Never - Seldom - Sometimes - Often - Always | |
|  | Does your work demand that you are good at coming up with new ideas? | - Never - Seldom - Sometimes - Often - Always | |
|  | Does your work require you to make difficult decisions? | - Never - Seldom - Sometimes - Often - Always | |
|  |  |  |  |

**EMOTIONAL DEMANDS**

|  | Does your work put you in emotionally disturbing situations? | - Never - Seldom - Sometimes - Often - Always | |
| --- | --- | --- | --- |
|  | Do you have to deal with other people’s personal problems as part of your work? | - Never - Seldom - Sometimes - Often - Always | |
|  | Is your work emotionally demanding? | - To a very small extent - To a small extent - Somewhat - To a large extent - To a very large extent | |
|  |  |  |  |

**INFLUENCE AT WORK**

|  | Do you have a large degree of influence on the decisions concerning your work? | - Never - Seldom - Sometimes - Often - Always | |
| --- | --- | --- | --- |
|  | Can you influence the amount of work assigned to you? | - Never - Seldom - Sometimes - Often - Always | |
|  | Do you have any influence on what you do at work? | - Never - Seldom - Sometimes - Often - Always | |
|  | Do you have any influence on HOW you do your work? | - Never - Seldom - Sometimes - Often - Always | |
|  |  |  |  |

**BULLYING**

|  | Have you been exposed to bullying at your workplace during the last 12 months? | - Yes, daily - Yes, weekly - Yes, monthly - Yes, a few times - Yes, once - No | |
| --- | --- | --- | --- |
|  |  |  |  |

**POSSIBILITIES FOR DEVELOPMENT**

|  | Do you have the possibility of learning new things through your work? | - To a very small extent - To a small extent - Somewhat - To a large extent - To a very large extent | |
| --- | --- | --- | --- |
|  | Can you use your skills or expertise in your work? | - To a very small extent - To a small extent - Somewhat - To a large extent - To a very large extent | |
|  |  |  |  |

**PREDICTABILITY**

|  | At your place of work, are you informed well in advance concerning for example important decisions, changes or plans for the future? | - To a very small extent - To a small extent - Somewhat - To a large extent - To a very large extent | |
| --- | --- | --- | --- |
|  | Do you receive all the information you need in order to do your work well? | - To a very small extent - To a small extent - Somewhat - To a large extent - To a very large extent | |
|  |  |  |  |

**ROLE CONFLICTS**

|  | Are contradictory demands placed on you at work? | - To a very small extent - To a small extent - Somewhat - To a large extent - To a very large extent | |
| --- | --- | --- | --- |
|  | Do you sometimes have to do things which ought to have been done in a different way? | - To a very small extent - To a small extent - Somewhat - To a large extent - To a very large extent | |
|  |  |  |  |

**SOCIAL SUPPORT FROM SUPERVISOR**

|  | How often is your immediate superior willing to listen to your problems at work, if needed? | - I do not have a supervisor - Never - Seldom - Sometimes - Often - Always | |
| --- | --- | --- | --- |
|  | How often do you get help and support from your immediate superior, if needed? | - I do not have a supervisor - Never - Seldom - Sometimes - Often - Always | |
|  |  |  |  |

**SOCIAL SUPPORT FROM COLLEGUES**

|  | How often do you get help and support from your colleagues, if needed? | - I do not have colleagues - Never - Seldom - Sometimes - Often - Always | |
| --- | --- | --- | --- |
|  | How often are your colleagues willing to listen to your problems at work, if needed? | - I do not have colleagues - Never - Seldom - Sometimes - Often - Always | |
|  | How often do your colleagues talk with you about how well you carry out your work? | - I do not have colleagues - Never - Seldom - Sometimes - Often - Always | |
|  |  |  |  |

**MEANING OF WORK**

|  | Is your work meaningful? | - To a very small extent - To a small extent - Somewhat - To a large extent - To a very large extent | |
| --- | --- | --- | --- |
|  | Do you feel that the work you do is important? | - To a very small extent - To a small extent - Somewhat - To a large extent - To a very large extent | |
|  |  |  |  |

**WORK ENGAGEMENT**

|  | The following 3 statements are about how you feel at work. If you have had this feeling, indicate how often you felt it. | | |
| --- | --- | --- | --- |
|  | At my work, I feel bursting with energy. | - Never - Seldom - Sometimes - Often - Always | |
|  | I am enthusiastic about my job | - Never - Seldom - Sometimes - Often - Always | |
|  | I am immersed in my work. | - Never - Seldom - Sometimes - Often - Always | |
|  |  |  |  |

**WORK LIFE CONFLICT**

|  | Do you feel that your work drains so much of your energy that it has a negative effect on your private life? | - To a very small extent - To a small extent - Somewhat - To a large extent - To a very large extent | |
| --- | --- | --- | --- |
|  | Do you feel that your work takes so much of your time that it has a negative effect on your private life? | - To a very small extent - To a small extent - Somewhat - To a large extent - To a very large extent | |
|  |  |  |  |

**BURNOUT (MBI: Emotional Exhaustion+Depersonalization)**

Questions of this section are concealed for copyright reasons. See Schaufeli WB, Bakker AB, Hoogduin K, Schaap C, Kladler A. On the clinical validity of the Maslach Burnout Inventory and the Burnout Measure. Psychol Health. 2001;16(5):565–82.

Use questions 01. 02. 03. 06. 08. 13. 14. 16. 20 to assess Emotional Exhaustion and questions 05. 10. 11. 15. 22 to assess Depersonalization

**SATISFACTION WITH WORK – JOB SATISFACTION**

|  | Regarding your work in general. How pleased are you with | | |
| --- | --- | --- | --- |
|  | your work prospects? | - Very unsatisfied - Unsatisfied - Neither/Not - Satisfied - Very satisfied | |
|  | the physical working conditions? | - Very unsatisfied - Unsatisfied - Neither/Not - Satisfied - Very satisfied | |
|  | the way your abilities are used? | - Very unsatisfied - Unsatisfied - Neither/Not - Satisfied - Very satisfied | |
|  | your job as a whole, everything taken into consideration? | - Very unsatisfied - Unsatisfied - Neither/Not - Satisfied - Very satisfied | |
|  | your salary? | - Very unsatisfied - Unsatisfied - Neither/Not - Satisfied - Very satisfied | |
|  |  |  |  |

**TURNOVER INTENTION**

|  | I intend to leave my current hospital for another one in the near future | - Strongly agree - Agree - Neither agree nor disagree - Disagree - Strongly disagree | |
| --- | --- | --- | --- |
|  | I intend to leave my healthcare profession for another job | - Strongly agree - Agree - Neither agree nor disagree - Disagree - Strongly disagree | |
|  |  |  |  |

**SUGGESTION FOR IMPROVEMENT OF THE METEOR PROJECT**

|  | From the list below, select the top three things you believe would be most effective in reducing burnout and improving well-being | - Limit physician work hours - Provide onsite child-care - Improve physician staffing levels - Improve nurse staffing levels - Reduce overnight call duties - Provide resilience training - Create time and places for meditation and reflection - Adequate fixed staffing ratio - Reduce overtime - Allow to spend more time with patients - Reduce bureaucracy and red tape - Reduce clinical documentation burden (paper-based/digital) - Other (write-in; provide statement on next page) | |
| --- | --- | --- | --- |
|  |  |  |  |

**Thank you for completing the METEOR survey!**

If you are interested in finding out more about our work and the “METEOR” project, please visit [www.meteorproject.eu](http://www.meteorproject.eu).

email: [survey@meteorproject.it](mailto:survey@meteorproject.it)

- 1. **Questionnaire for nurses: current work experience**

Dear Madam / Sir,

We appreciate your participation in this survey regarding the **working conditions, general health and well-being of nurses.** METEOR is a project funded by the European Union’s Health Programme (2014 - 2020) (Grant Agreement 101018310). METEOR's mission is to improve the mental health and well-being of European health workers.

We would like to ask you to respond to a **series of questions** on your perceptions of various areas of your work environment, such as your relationship with other health professionals, your workload and staffing and your intention to leave the profession (if any).

The questionnaire should be completed **within three weeks** after getting this invitation and it should take approximately **15 minutes** to complete. As a nurse with firsthand knowledge of your hospital, your replies are critical and will be used to improve patient care and your work environment.

Your responses to the questions are **strictly personal and confidential.** Each question will require a response. Kindly select the option that most accurately expresses your current feelings or opinions about the subject. During the three-week survey period, you may access the survey at any time and at your convenience. You are only permitted to complete the survey once.

Kind regards,

*[Contact details of each partner according to the country where the survey is submitted]*

**ABOUT YOU**

|  | Gender | - Male - Female - Other |
| --- | --- | --- |
|  | Age | …………………….. |
|  | How would you describe your current living situation? | - Living alone - Living together with partner - Living with partner and child(ren), - Living with friends / housemates - Living with parents - Other…................. |
|  | Highest achieved educational level | - Bachelor degree - Master degree - PhD |

**Work context**

|  | Home Hospital | | - UZ Leuven Academic hospital, Belgium - AZ Delta, Belgium - Medical University of Silesia, Poland - SP ZOZ Szpital Wielospecjalistyczny w Jaworznie, Poland - Amsterdam University Medical Centre, the Netherlands - Spaarne Gasthuis, the Netherlands - Palermo University Hospital, Italy - Local Health Facility of Agrigento, Italy | | |
| --- | --- | --- | --- | --- | --- |
|  | Form of employment | | - Full-time - Part-time (anything <100%) | | |
|  | Type of contract | | - Permanent - Non-permanent (temporary) | | |
|  | How many years have you been working in this hospital? | | - <=1 year - 2 years - … - >40 | | |
|  | Do you do nightshifts? | | - Yes - No | | |
|  | Specialty area (please, consider your current job) | | - Anaesthesia and resuscitation - Cardiology - Dermatology and venereology - Physical Medicine & Rehabilitation - Gastroenterology - Forensic Medicine - Geriatrics - Gynaecology and obstetrics - General Surgery - Internal Medicine - Child and adolescent psychiatry - Psychiatry - Clinical biology - Oncology - Neurosurgery - Neurology - Ophthalmology - Nuclear Medicine - Ophthalmology - Orthopaedic Surgery - Otorhinolaryngology, head and neck surgery - Pathological anatomy - Paediatrics - Plastic, reconstructive and aesthetic surgery - Pneumology - Radiotherapy - Rheumatology - Radiology - Stomatology, Oral and Maxillofacial Surgery - Emergency Medicine - Urology - Occupational Medicine - General Practice - Hygiene and preventive medicine - Other… (specify) | | |
|  | Have you been involved as frontline with Covid-19 patients? | | - Yes - If No, goes to the next section | | |
|  | How long did you work as frontline with Covid-19 patients? | | - Less or equal than 1 year - More than 1 year | | |
|  |  |  | |  |  |

**Working conditions**

|  | The hospital provides all the materials I need to do my job at best | - Strongly agree - Agree - Neither agree nor disagree - Disagree - Strongly disagree | |
| --- | --- | --- | --- |
|  |  |  |  |

**Health problems during your last job**

|  | Have you suffered from any illness, disability or other physical or mental problem caused or made worse by work, apart from the occupational injuries, in your last job in the past three years? | - Yes - No | |
| --- | --- | --- | --- |
|  |  |  |  |

**STAFFING**

|  | We have enough staff to handle the workload | - Strongly agree - Agree - Neither agree nor disagree - Disagree - Strongly disagree | |
| --- | --- | --- | --- |
|  | Staff in this unit work longer hours than is best for patient care. | - Strongly disagree - Disagree - Neither agree nor disagree - Agree - Strongly agree | |
|  | We use more temporary staff than is best for patient care. | - Strongly disagree - Disagree - Neither agree nor disagree - Agree - Strongly agree | |
|  | We work in "crisis mode" trying to do too much, too quickly. | - Strongly disagree - Disagree - Neither agree nor disagree - Agree - Strongly agree | |
|  |  |  |  |

**WORK PACE**

|  | Do you have to work very fast? | - Never - Seldom - Sometimes - Often - Always | |
| --- | --- | --- | --- |
|  | Do you work at a high pace throughout the day? | - To a very small extent - To a small extent - Somewhat - To a large extent - To a very large extent | |
|  |  |  |  |

**QUANTITATIVE DEMANDS**

|  | Is your workload unevenly distributed so it piles up? | - Never - Seldom - Sometimes - Often - Always | |
| --- | --- | --- | --- |
|  | How often do you not have time to complete all your work tasks? | - Never - Seldom - Sometimes - Often - Always | |
|  | Do you get behind with your work? | - Never - Seldom - Sometimes - Often - Always | |
|  | Do you have enough time for your work tasks? | - Always - Often - Sometimes - Seldom - Never | |
|  |  |  |  |

**COGNITIVE DEMANDS**

|  | Do you have to keep your eyes on lots of things while you work? | - Never - Seldom - Sometimes - Often - Always | |
| --- | --- | --- | --- |
|  | Does your work require that you remember at lot of things? | - Never - Seldom - Sometimes - Often - Always | |
|  | Does your work demand that you are good at coming up with new ideas? | - Never - Seldom - Sometimes - Often - Always | |
|  | Does your work require you to make difficult decisions? | - Never - Seldom - Sometimes - Often - Always | |
|  |  |  |  |

**EMOTIONAL DEMANDS**

|  | Does your work put you in emotionally disturbing situations? | - Never - Seldom - Sometimes - Often - Always | |
| --- | --- | --- | --- |
|  | Do you have to deal with other people’s personal problems as part of your work? | - Never - Seldom - Sometimes - Often - Always | |
|  | Is your work emotionally demanding? | - To a very small extent - To a small extent - Somewhat - To a large extent - To a very large extent | |
|  |  |  |  |

**INFLUENCE AT WORK**

|  | Do you have a large degree of influence on the decisions concerning your work? | - Never - Seldom - Sometimes - Often - Always | |
| --- | --- | --- | --- |
|  | Can you influence the amount of work assigned to you? | - Never - Seldom - Sometimes - Often - Always | |
|  | Do you have any influence on what you do at work? | - Never - Seldom - Sometimes - Often - Always | |
|  | Do you have any influence on HOW you do your work? | - Never - Seldom - Sometimes - Often - Always | |
|  |  |  |  |

**BULLYING**

|  | Have you been exposed to bullying at your workplace during the last 12 months? | - Yes, daily - Yes, weekly - Yes, monthly - Yes, a few times - Yes, once - No | |
| --- | --- | --- | --- |
|  |  |  |  |

**POSSIBILITIES FOR DEVELOPMENT**

|  | Do you have the possibility of learning new things through your work? | - To a very small extent - To a small extent - Somewhat - To a large extent - To a very large extent | |
| --- | --- | --- | --- |
|  | Can you use your skills or expertise in your work? | - To a very small extent - To a small extent - Somewhat - To a large extent - To a very large extent | |
|  |  |  |  |

**PREDICTABILITY**

|  | At your place of work, are you informed well in advance concerning for example important decisions, changes or plans for the future? | - To a very small extent - To a small extent - Somewhat - To a large extent - To a very large extent | |
| --- | --- | --- | --- |
|  | Do you receive all the information you need in order to do your work well? | - To a very small extent - To a small extent - Somewhat - To a large extent - To a very large extent | |
|  |  |  |  |

**ROLE CONFLICTS**

|  | Are contradictory demands placed on you at work? | - To a very small extent - To a small extent - Somewhat - To a large extent - To a very large extent | |
| --- | --- | --- | --- |
|  | Do you sometimes have to do things which ought to have been done in a different way? | - To a very small extent - To a small extent - Somewhat - To a large extent - To a very large extent | |
|  |  |  |  |

**SOCIAL SUPPORT FROM SUPERVISOR**

|  | How often is your immediate superior willing to listen to your problems at work, if needed? | - I do not have a supervisor - Never - Seldom - Sometimes - Often - Always | |
| --- | --- | --- | --- |
|  | How often do you get help and support from your immediate superior, if needed? | - I do not have a supervisor - Never - Seldom - Sometimes - Often - Always | |
|  |  |  |  |

**SOCIAL SUPPORT FROM COLLEGUES**

|  | How often do you get help and support from your colleagues, if needed? | - I do not have colleagues - Never - Seldom - Sometimes - Often - Always | |
| --- | --- | --- | --- |
|  | How often are your colleagues willing to listen to your problems at work, if needed? | - I do not have colleagues - Never - Seldom - Sometimes - Often - Always | |
|  | How often do your colleagues talk with you about how well you carry out your work? | - I do not have colleagues - Never - Seldom - Sometimes - Often - Always | |
|  |  |  |  |

**MEANING OF WORK**

|  | Is your work meaningful? | - To a very small extent - To a small extent - Somewhat - To a large extent - To a very large extent | |
| --- | --- | --- | --- |
|  | Do you feel that the work you do is important? | - To a very small extent - To a small extent - Somewhat - To a large extent - To a very large extent | |
|  |  |  |  |

**WORK ENGAGEMENT**

|  | The following 3 statements are about how you feel at work. If you have had this feeling, indicate how often you felt it. | | |
| --- | --- | --- | --- |
|  | At my work, I feel bursting with energy. | - Never - Seldom - Sometimes - Often - Always | |
|  | I am enthusiastic about my job | - Never - Seldom - Sometimes - Often - Always | |
|  | I am immersed in my work. | - Never - Seldom - Sometimes - Often - Always | |
|  |  |  |  |

**WORK LIFE CONFLICT**

|  | Do you feel that your work drains so much of your energy that it has a negative effect on your private life? | - To a very small extent - To a small extent - Somewhat - To a large extent - To a very large extent | |
| --- | --- | --- | --- |
|  | Do you feel that your work takes so much of your time that it has a negative effect on your private life? | - To a very small extent - To a small extent - Somewhat - To a large extent - To a very large extent | |
|  |  |  |  |

**BURNOUT (MBI: Emotional Exhaustion+Depersonalization)**

Questions of this section are concealed for copyright reasons. See Schaufeli WB, Bakker AB, Hoogduin K, Schaap C, Kladler A. On the clinical validity of the Maslach Burnout Inventory and the Burnout Measure. Psychol Health. 2001;16(5):565–82.

Use questions 01. 02. 03. 06. 08. 13. 14. 16. 20 to assess Emotional Exhaustion and questions 05. 10. 11. 15. 22 to assess Depersonalization

**SATISFACTION WITH WORK – JOB SATISFACTION**

|  | Regarding your work in general. How pleased are you with | | |
| --- | --- | --- | --- |
|  | your work prospects? | - Very unsatisfied - Unsatisfied - Neither/Not - Satisfied - Very satisfied | |
|  | the physical working conditions? | - Very unsatisfied - Unsatisfied - Neither/Not - Satisfied - Very satisfied | |
|  | the way your abilities are used? | - Very unsatisfied - Unsatisfied - Neither/Not - Satisfied - Very satisfied | |
|  | your job as a whole, everything taken into consideration? | - Very unsatisfied - Unsatisfied - Neither/Not - Satisfied - Very satisfied | |
|  | your salary? | - Very unsatisfied - Unsatisfied - Neither/Not - Satisfied - Very satisfied | |
|  |  |  |  |

**TURNOVER INTENTION**

|  | I intend to leave my current hospital for another one in the near future | - Strongly agree - Agree - Neither agree nor disagree - Disagree - Strongly disagree | |
| --- | --- | --- | --- |
|  | I intend to leave my healthcare profession for another job | - Strongly agree - Agree - Neither agree nor disagree - Disagree - Strongly disagree | |
|  |  |  |  |

**SUGGESTION FOR IMPROVEMENT OF THE METEOR PROJECT**

|  | From the list below, select the top three things you believe would be most effective in reducing burnout and improving well-being | - Limit physician work hours - Provide onsite child-care - Improve physician staffing levels - Improve nurse staffing levels - Reduce overnight call duties - Provide resilience training - Create time and places for meditation and reflection - Adequate fixed staffing ratio - Reduce overtime - Allow to spend more time with patients - Reduce bureaucracy and red tape - Reduce clinical documentation burden (paper-based/digital) - Other (write-in; provide statement on next page) | |
| --- | --- | --- | --- |
|  |  |  |  |

**Thank you for completing the METEOR survey!**

If you are interested in finding out more about our work and the “METEOR” project, please visit [www.meteorproject.eu](http://www.meteorproject.eu).

email: [survey@meteorproject.it](mailto:survey@meteorproject.it)

1. **Italian questionnaire**

**2.1 Questionario per medici: esperienza di lavoro attuale**

Gentile dottore/dottoressa

La ringraziamo per aver accettato di prender parte a questa ricerca sulle condizioni di lavoro, la salute generale e il benessere dei medici. METEOR è un progetto finanziato dal Programma per la salute dell’Unione europea (2014-2020) (Accordo n. 101018310), che si propone di migliorare la salute mentale e il benessere degli operatori sanitari in Europa. A questo scopo, le chiederemo di rispondere ad una serie di domande che riguardano il suo ambiente di lavoro, il clima interno al reparto e all’ospedale, nonché la soddisfazione rispetto al proprio lavoro e l’eventuale intenzione di lasciarlo. In qualità di medico con una conoscenza diretta del suo ospedale, le sue risposte sono fondamentali e verranno utilizzate per migliorare l’assistenza ai pazienti e il suo ambiente di lavoro. Potrà partecipare alla ricerca una sola volta.

Il questionario dovrà essere completato entro tre settimane dalla ricezione dell’invito e in questo periodo potrà accedere al sondaggio in qualsiasi momento e a suo piacimento. Il completamento del questionario richiederà circa 15 minuti. Le sue risposte alle domande sono strettamente personali e confidenziali. Ogni domanda richiederà una risposta. Selezioni l’opzione che esprime più accuratamente le sue sensazioni o opinioni attuali sull’argomento.

Cordiali saluti,

**A PROPOSITO DI LEI**

|  | Genere | - Uomo - Donna - Altro |
| --- | --- | --- |
|  | Età | ………………….. |
|  | Come descriverebbe la sua attuale condizione di vita? | - Vivo da solo - Convivo con il partner - Vivo con il partner e i figlio/ figli - Vivo con gli amici / coinquilini - Vivo con I genitori - Altro (specificare)…….............. |

**Contesto lavorativo**

|  | Nome dell’Ospedale | - Ospedale Universitario di UZ Leuven - AZ Delta, Belgio - Ospedale Jaworznie - Ospedale Universitario di Silesia - Centro medico Universitario di Amsterdam - Spaarne Gasthuis, Olanda - Ospedale Universitario di Palermo - ASL di Agrigento | |
| --- | --- | --- | --- |
|  | Tipo di impiego. | - Full-time - Part-time (qualsiasi tipologia <100%) | |
|  | Tipo di contratto | - Indeterminato - Determinato (temporaneo) | |
|  | Quanti anni ha lavorato in questo ospedale? | - <=1 anno - 2 anni - … - >40 | |
|  | Fa turni notturni? | - Yes - No | |
|  | Area di specializzazione (per favore, consideri il suo attuale lavoro) | - Anestesia e rianimazione - Cardiologia - Dermatologia e venereologia - Medicina fisica e riabilitazione - Gastroenterologia - Medicina Forense - Geriatria - Ginecologia ed ostetricia - Chirurgia Generale - Medicina Interna - Neuropsichiatria Infantile - Psichiatria - Biologia Clinica - Oncologia - Neurochirurgia - Neurologia - Oftalmologia - Medicina Nucleare - Chirurgia Ortopedica - Otorinolaringoiatria e chirurgia del collo e della testa - Anatomia Patologica - Pediatria - Chirurgia plastica, estetica e ricostruttiva - Pneumologia - Radioterapia - Reumatologia - Radiologia - Chirurgia maxilla-facciale - Medicina d’ Urgenza - Urologia - Medicina del lavoro - Medicina Generale - Igiene e medicina apportve - Altro… (specificare) | |
|  | Ha lavorato in prima linea con i pazienti Covid-19? | - Sì - Se No, vai alla sezione successiva | |
|  | Per quanto tempo ha lavorato in prima linea con i pazienti Covid-19? | - <=1anno - Più di 1 anno | |
|  |  |  |  |

**condiZIONI LAVORATIVE**

|  | L’ospedale fornisce tutto il materiale di cui ho bisogno per svolgere al meglio il mio lavoro | - Fortemente d’accordo - D’accordo - Né d’accordo né in disaccordo - In Disaccordo - Fortemente in disaccordo | |
| --- | --- | --- | --- |
|  |  |  |  |

**PROBLEMI DI SALUTE DURANTE IL TUO ULTIMO IMPIEGO**

|  | Ha sofferto di malattie, disabilità o altri problemi fisici o mentali causati o aggravati dal lavoro, a parte gli infortuni, nel suo ultimo lavoro negli ultimi tre anni? | - Sì - No | |
| --- | --- | --- | --- |
|  |  |  |  |

**PERSONALE**

|  | Abbiamo abbastanza personale per gestire il carico di lavoro | - Fortemente in disaccordo - In Disaccordo - Né d’accordo né in disaccordo - D’accordo - Fortemente d’accordo | |
| --- | --- | --- | --- |
|  | Il personale di questa unità lavora più a lungo di quanto sia meglio per la cura del paziente. | - Fortemente in disaccordo - In Disaccordo - Né d’accordo né in disaccordo - D’accordo - Fortemente d’accordo | |
|  | Usiamo più personale temporaneo di quanto sia meglio per la cura del paziente. | - Fortemente in disaccordo - In Disaccordo - Né d’accordo né in disaccordo - D’accordo - Fortemente d’accordo | |
|  | Lavoriamo in “modalità crisi” cercando di fare troppo, troppo velocemente. | - Fortemente in disaccordo - In Disaccordo - Né d’accordo né in disaccordo - D’accordo - Fortemente d’accordo | |
|  |  |  |  |

**LUOGO DI LAVORO**

|  | Deve lavorare molto velocemente? | - Mai - Raramente - A volte - Spesso - Sempre | |
| --- | --- | --- | --- |
|  | Lavora ad un ritmo elevato durante il giorno? | - In misura molto ridotta - In apport parte - Un po’ - In larga misura - In misura molto larga | |
|  |  |  |  |

**RICHIESTE QUANTITATIVE**

|  | Il suo carico di lavoro è distribuito in modo non uniforme e si accumula? | - Mai - Raramente - A volte - Spesso - Sempre | |
| --- | --- | --- | --- |
|  | Quante volte non ha tempo per completare tutte le sue attività lavorative? | - Mai - Raramente - A volte - Spesso - Sempre | |
|  | Rimane indietro con il suo lavoro? | - Mai - Raramente - A volte - Spesso - Sempre | |
|  | Ha abbastanza tempo per le sue attività lavorative? | - Mai - Raramente - A volte - Spesso - Sempre | |
|  |  |  |  |

**RICHIESTE COGNITIVE**

|  | Deve tenere d’occhio molte cose mentre lavora? | - Mai - Raramente - A volte - Spesso - Sempre | |
| --- | --- | --- | --- |
|  | Il suo lavoro richiede che ricordi molte cose? | - Mai - Raramente - A volte - Spesso - Sempre | |
|  | Il suo lavoro richiede che lei sia bravo a trovare nuove idee? | - Mai - Raramente - A volte - Spesso - Sempre | |
|  | Il suo lavoro le richiede di prendere decisioni difficili? | - Mai - Raramente - A volte - Spesso - Sempre | |
|  |  |  |  |

**RICHIESTE EMOTIVE**

|  | Il suo lavoro la mette in situazioni emotivamente disturbanti? | - Mai - Raramente - A volte - Spesso - Sempre | |
| --- | --- | --- | --- |
|  | Deve affrontare i problemi personali di altre persone come parte del suo lavoro? | - Mai - Raramente - A volte - Spesso - Sempre | |
|  | Il suo lavoro è emotivamente impegnativo? | - In misura molto ridotta - In apport parte - Un po’ - In larga misura - In misura molto larga | |
|  |  |  |  |

**INFLUENZA SUL LAVORO**

|  | Ha un grande grado di influenza sulle decisioni riguardanti il suo lavoro? | - Mai - Raramente - A volte - Spesso - Sempre | |
| --- | --- | --- | --- |
|  | Può influenzare la quantità di lavoro che le è stata assegnata? | - Mai - Raramente - A volte - Spesso - Sempre | |
|  | Ha qualche influenza su quello che fa al lavoro? | - Mai - Raramente - A volte - Spesso - Sempre | |
|  | Ha qualche influenza su COME svolge il suo lavoro? | - Mai - Raramente - A volte - Spesso - Sempre | |
|  |  |  |  |

**BULLISMO**

|  | È stato esposto ad episodi di bullismo sul posto di lavoro negli ultimi 12 mesi? | - Sì, tutti i giorni - Sì, settimanalmente - Sì, mensilmente - Sì, un paio di volte - Sì, una volta - No | |
| --- | --- | --- | --- |
|  |  |  |  |

**POSSIBILITÀ DI SVILUPPO**

|  | Ha la possibilità di imparare cose nuove attraverso il suo lavoro? | - In misura molto ridotta - In apport parte - Un po’ - In larga misura - In misura molto larga | |
| --- | --- | --- | --- |
|  | Può usare le sue abilità o competenze nel suo lavoro? | - In misura molto ridotta - In apport parte - Un po’ - In larga misura - In misura molto larga | |
|  |  |  |  |

**PREVEDIBILITÀ**

|  | Sul suo posto di lavoro, è informato con largo anticipo riguardo ad esempio a decisioni importanti, cambiamenti o piani per il futuro? | - In misura molto ridotta - In apport parte - Un po’ - In larga misura - In misura molto larga | |
| --- | --- | --- | --- |
|  | Riceve tutte le informazioni di cui ha bisogno per svolgere bene il suo lavoro? | - In misura molto ridotta - In apport parte - Un po’ - In larga misura - In misura molto larga | |
|  |  |  |  |

**CONFLITTO DI RUOLO**

|  | Le vengono poste richieste contraddittorie al lavoro? | - In misura molto ridotta - In apport parte - Un po’ - In larga misura - In misura molto larga | |
| --- | --- | --- | --- |
|  | A volte deve fare cose che avrebbero dovuto essere fatte in un modo diverso? | - In misura molto ridotta - In apport parte - Un po’ - In larga misura - In misura molto larga | |
|  |  |  |  |

**SUPPORTO SOCIALE DA PARTE DEL SUPERVISORE**

|  | Con quale frequenza il suo diretto superiore è disposto ad ascoltare i suoi problemi sul lavoro, se necessario? | - Non ho un supervisore - Mai - Raramente - A volte - Spesso - Sempre | |
| --- | --- | --- | --- |
|  | Con quale frequenza riceve aiuto e supporto dal suo diretto superiore, se necessario? | - Non ho un supervisore - Mai - Raramente - A volte - Spesso - Sempre | |
|  |  |  |  |

**SUPPORTO SOCIALE DA PARTE DEI COLLEGHI**

|  | Con quale frequenza riceve aiuto e supporto dai suoi colleghi, se necessario? | - Non ho colleghi - Mai - Raramente - A volte - Spesso - Sempre | |
| --- | --- | --- | --- |
|  | Con quale frequenza i suoi colleghi sono disposti ad ascoltare i suoi problemi sul lavoro, se necessario? | - Non ho colleghi - Mai - Raramente - A volte - Spesso - Sempre | |
|  | Con quale frequenza i suoi colleghi le parlano di come svolge il suo lavoro? | - Non ho colleghi - Mai - Raramente - A volte - Spesso - Sempre | |
|  |  |  |  |

**SENSO DEL LAVORO**

|  | Il suo lavoro è significativo? | - In misura molto ridotta - In apport parte - Un po’ - In larga misura - In misura molto larga | |
| --- | --- | --- | --- |
|  | Sente che il lavoro che fa è importante? | - In misura molto ridotta - In apport parte - Un po’ - In larga misura - In misura molto larga | |
|  |  |  |  |

**IMPEGNO LAVORATIVO**

|  | Le seguenti affermazioni riguardano come si sente al lavoro. Se ha avuto questa sensazione, indichi quanto spesso l’ha provata. | | |
| --- | --- | --- | --- |
|  | Al lavoro, mi sento scoppiare di energia. | - Mai - Raramente - A volte - Spesso - Sempre | |
|  | Sono entusiasta del mio lavoro. | - Mai - Raramente - A volte - Spesso - Sempre | |
|  | Sono immerso nel mio lavoro | - Mai - Raramente - A volte - Spesso - Sempre | |
|  |  |  |  |

**CONFLITTO NELLA VITA LAVORATIVA**

|  | Ritiene che il suo lavoro prosciughi così tanto la sua energia da avere un effetto negativo sulla sua vita privata? | - In misura molto ridotta - In apport parte - Un po’ - In larga misura - In misura molto larga | |
| --- | --- | --- | --- |
|  | Ritiene che il suo lavoro richieda così tanto tempo da avere un effetto negativo sulla sua vita privata? | - In misura molto ridotta - In apport parte - Un po’ - In larga misura - In misura molto larga | |
|  |  |  |  |

**BURNOUT (MBI: Emotional Exhaustion+Depersonalization)**

Questions of this section are concealed for copyright reasons. See Schaufeli WB, Bakker AB, Hoogduin K, Schaap C, Kladler A. On the clinical validity of the Maslach Burnout Inventory and the Burnout Measure. Psychol Health. 2001;16(5):565–82.

Use questions 01. 02. 03. 06. 08. 13. 14. 16. 20 to assess Emotional Exhaustion and questions 05. 10. 11. 15. 22 to assess Depersonalization

**SODDISFAZIONE LAVORATIVA**

|  | Riguardo il suo lavoro in generale. Quanto è soddisfatto | | |
| --- | --- | --- | --- |
|  | Delle sue prospettive lavorative? | - Veramente insoddisfatto - Insoddisfatto - Né insoddisfatto né soddisfatto - Soddisfatto - Veramente soddisfatto | |
|  | Delle sue condizioni fisiche di lavoro? | - Veramente insoddisfatto - Insoddisfatto - Né insoddisfatto né soddisfatto - Soddisfatto - Veramente soddisfatto | |
|  | Del modo in cui vengono utilizzate le sue abilità? | - Veramente insoddisfatto - Insoddisfatto - Né insoddisfatto né soddisfatto - Soddisfatto - Veramente soddisfatto | |
|  | Del suo lavoro nel suo insieme, prendendo in considerazione tutto? | - Veramente insoddisfatto - Insoddisfatto - Né insoddisfatto né soddisfatto - Soddisfatto - Veramente soddisfatto | |
|  | Del suo stipendio? | - Veramente insoddisfatto - Insoddisfatto - Né insoddisfatto né soddisfatto - Soddisfatto - Veramente soddisfatto | |
|  |  |  |  |

**INCLINAZIONE A CERCARE UN NUOVO POSTO DI LAVORO**

|  | Ho intenzione di lasciare il mio attuale ospedale per un altro nel prossimo futuro | - Fortemente d’accordo - D’accordo - Né d’accordo né in disaccordo - In Disaccordo - Fortemente in disaccordo | |
| --- | --- | --- | --- |
|  | Ho intenzione di lasciare la mia professione sanitaria per un altro lavoro | - Fortemente d’accordo - D’accordo - Né d’accordo né in disaccordo - In Disaccordo - Fortemente in disaccordo | |
|  |  |  |  |

**SUGGERIMENTI PER MIGLIORARE IL PROGETTO METEOR**

|  | Dall’elenco sottostante, selezioni le tre cose principali che ritiene siano più efficaci nel ridurre il burnout e nel migliorare il benessere | - Limitare l’orario di lavoro dei medici - Fornire assistenza all’infanzia in loco - Migliorare il numero di personale medico - Migliorare il numero di personale infermieristico - Ridurre le chiamate notturne - Fornire formazione sulla resilienza - Creazione di tempo e luoghi per la meditazione e la riflessione - Adeguato apport personale/paziente - Ridurre gli straordinari - Consentire di trascorrere più tempo con i pazienti - Ridurre la burocrazia - Ridurre il carico di documentazione clinica (cartaceo/digitale) - Altro (scrivere; fornire una dichiarazione nella pagina successiva) | |
| --- | --- | --- | --- |
|  |  |  |  |

**Grazie per aver completato il questionario METEOR!**

Se vuole saperne di più sul nostro lavoro e sul progetto “METEOR”, visiti [www.meteorproject.eu](http://www.meteorproject.eu).

Email: [survey@meteorproject.eu](mailto:survey@meteorproject.eu)

**2.2 Questionario per infermieri: esperienza di lavoro attuale**

Gentile dottore/dottoressa

La ringraziamo per aver accettato di prender parte a questa ricerca sulle condizioni di lavoro, la salute generale e il benessere degli infermieri. METEOR è un progetto finanziato dal Programma per la salute dell'Unione europea (2014-2020) (Accordo n. 101018310), che si propone di migliorare la salute mentale e il benessere degli operatori sanitari in Europa. A questo scopo, le chiederemo di rispondere ad una serie di domande che riguardano il suo ambiente di lavoro, il clima interno al reparto e all’ospedale, nonché la soddisfazione rispetto al proprio lavoro e l’eventuale intenzione di lasciarlo. In qualità di infermiere con una conoscenza diretta del suo ospedale, le sue risposte sono fondamentali e verranno utilizzate per migliorare l'assistenza ai pazienti e il suo ambiente di lavoro. Potrà partecipare alla ricerca una sola volta.

Il questionario dovrà essere completato entro tre settimane dalla ricezione dell'invito e in questo periodo potrà accedere al sondaggio in qualsiasi momento e a suo piacimento. Il completamento del questionario richiederà circa 15 minuti. Le sue risposte alle domande sono strettamente personali e confidenziali. Ogni domanda richiederà una risposta. Selezioni l'opzione che esprime più accuratamente le sue sensazioni o opinioni attuali sull'argomento.

Cordiali saluti,

**A PROPOSITO DI LEI**

|  | Genere | - Uomo - Donna - Altro |
| --- | --- | --- |
|  | Età | …………………….. |
|  | Come descriverebbe la sua attuale condizione di vita? | - Vivo da solo - Convivo con il partner - Vivo con il partner e i figlio/ figli - Vivo con gli amici / coinquilini - Vivo con I genitori - Altro (specificare)…............... |
|  | Livello di istruzione più elevato | - Laurea triennale - Laurea magistrale - Dottorato di Ricerca |

**Contesto lavorativo**

|  | Nome dell’Ospedale | - Ospedale Universitario di UZ Leuven - AZ Delta, Belgio - Ospedale Jaworznie - Ospedale Universitario di Silesia - Centro medico Universitario di Amsterdam - Spaarne Gasthuis, Olanda - Ospedale Universitario di Palermo - ASL di Agrigento | |
| --- | --- | --- | --- |
|  | Tipo di impiego. | - Full-time - Part-time (qualsiasi tipologia <100%) | |
|  | Tipo di contratto | - Indeterminato - Determinato (temporaneo) | |
|  | Quanti anni ha lavorato in questo ospedale? | - <=1 anno - 2 anni - … - >40 | |
|  | Fa turni notturni? | - Yes - No | |
|  | Area di specializzazione  (per favore, consideri il suo attuale lavoro) | - Anestesia e rianimazione - Cardiologia - Dermatologia e venereologia - Medicina fisica e riabilitazione - Gastroenterologia - Medicina Forense - Geriatria - Ginecologia ed ostetricia - Chirurgia Generale - Medicina Interna - Neuropsichiatria Infantile - Psichiatria - Biologia Clinica - Oncologia - Neurochirurgia - Neurologia - Oftalmologia - Medicina Nucleare - Chirurgia Ortopedica - Otorinolaringoiatria e chirurgia del collo e della testa - Anatomia Patologica - Pediatria - Chirurgia plastica, estetica e ricostruttiva - Pneumologia - Radioterapia - Reumatologia - Radiologia - Chirurgia maxilla-facciale - Medicina d’ Urgenza - Urologia - Medicina del lavoro - Medicina Generale - Igiene e medicina preventiva - Altro… (specificare) | |
|  | Ha lavorato in prima linea con i pazienti Covid-19? | - Sì - Se No, vai alla sezione successiva | |
|  | Per quanto tempo ha lavorato in prima linea con i pazienti Covid-19? | - <=1anno - Più di 1 anno | |
|  |  |  |  |

**condiZIONI LAVORATIVE**

|  | L'ospedale fornisce tutto il materiale di cui ho bisogno per svolgere al meglio il mio lavoro | - Fortemente d’accordo - D’accordo - Né d’accordo né in disaccordo - In Disaccordo - Fortemente in disaccordo | |
| --- | --- | --- | --- |
|  |  |  |  |

**PROBLEMI DI SALUTE DURANTE IL TUO ULTIMO IMPIEGO**

|  | Ha sofferto di malattie, disabilità o altri problemi fisici o mentali causati o aggravati dal lavoro, a parte gli infortuni, nel suo ultimo lavoro negli ultimi tre anni? | - Sì - No | |
| --- | --- | --- | --- |
|  |  |  |  |

**PERSONALE**

|  | Abbiamo abbastanza personale per gestire il carico di lavoro | - Fortemente in disaccordo - In Disaccordo - Né d’accordo né in disaccordo - D’accordo - Fortemente d’accordo | |
| --- | --- | --- | --- |
|  | Il personale di questa unità lavora più a lungo di quanto sia meglio per la cura del paziente. | - Fortemente in disaccordo - In Disaccordo - Né d’accordo né in disaccordo - D’accordo - Fortemente d’accordo | |
|  | Usiamo più personale temporaneo di quanto sia meglio per la cura del paziente. | - Fortemente in disaccordo - In Disaccordo - Né d’accordo né in disaccordo - D’accordo - Fortemente d’accordo | |
|  | Lavoriamo in "modalità crisi" cercando di fare troppo, troppo velocemente. | - Fortemente in disaccordo - In Disaccordo - Né d’accordo né in disaccordo - D’accordo - Fortemente d’accordo | |
|  |  |  |  |

**LUOGO DI LAVORO**

|  | Deve lavorare molto velocemente? | - Mai - Raramente - A volte - Spesso - Sempre | |
| --- | --- | --- | --- |
|  | Lavora ad un ritmo elevato durante il giorno? | - In misura molto ridotta - In piccola parte - Un po' - In larga misura - In misura molto larga | |
|  |  |  |  |

**RICHIESTE QUANTITATIVE**

|  | Il suo carico di lavoro è distribuito in modo non uniforme e si accumula? | - Mai - Raramente - A volte - Spesso - Sempre | |
| --- | --- | --- | --- |
|  | Quante volte non ha tempo per completare tutte le sue attività lavorative? | - Mai - Raramente - A volte - Spesso - Sempre | |
|  | Rimane indietro con il suo lavoro? | - Mai - Raramente - A volte - Spesso - Sempre | |
|  | Ha abbastanza tempo per le sue attività lavorative? | - Mai - Raramente - A volte - Spesso - Sempre | |
|  |  |  |  |

**RICHIESTE COGNITIVE**

|  | Deve tenere d'occhio molte cose mentre lavora? | - Mai - Raramente - A volte - Spesso - Sempre | |
| --- | --- | --- | --- |
|  | Il suo lavoro richiede che ricordi molte cose? | - Mai - Raramente - A volte - Spesso - Sempre | |
|  | Il suo lavoro richiede che lei sia bravo a trovare nuove idee? | - Mai - Raramente - A volte - Spesso - Sempre | |
|  | Il suo lavoro le richiede di prendere decisioni difficili? | - Mai - Raramente - A volte - Spesso - Sempre | |
|  |  |  |  |

**RICHIESTE EMOTIVE**

|  | Il suo lavoro la mette in situazioni emotivamente disturbanti? | - Mai - Raramente - A volte - Spesso - Sempre | |
| --- | --- | --- | --- |
|  | Deve affrontare i problemi personali di altre persone come parte del suo lavoro? | - Mai - Raramente - A volte - Spesso - Sempre | |
|  | Il suo lavoro è emotivamente impegnativo? | - In misura molto ridotta - In piccola parte - Un po' - In larga misura - In misura molto larga | |
|  |  |  |  |

**INFLUENZA SUL LAVORO**

|  | Ha un grande grado di influenza sulle decisioni riguardanti il suo lavoro? | - Mai - Raramente - A volte - Spesso - Sempre | |
| --- | --- | --- | --- |
|  | Può influenzare la quantità di lavoro che le è stata assegnata? | - Mai - Raramente - A volte - Spesso - Sempre | |
|  | Ha qualche influenza su quello che fa al lavoro? | - Mai - Raramente - A volte - Spesso - Sempre | |
|  | Ha qualche influenza su COME svolge il suo lavoro? | - Mai - Raramente - A volte - Spesso - Sempre | |
|  |  |  |  |

**BULLISMO**

|  | È stato esposto ad episodi di bullismo sul posto di lavoro negli ultimi 12 mesi? | - Sì, tutti i giorni - Sì, settimanalmente - Sì, mensilmente - Sì, un paio di volte - Sì, una volta - No | |
| --- | --- | --- | --- |
|  |  |  |  |

**POSSIBILITÀ DI SVILUPPO**

|  | Ha la possibilità di imparare cose nuove attraverso il suo lavoro? | - In misura molto ridotta - In piccola parte - Un po' - In larga misura - In misura molto larga | |
| --- | --- | --- | --- |
|  | Può usare le sue abilità o competenze nel suo lavoro? | - In misura molto ridotta - In piccola parte - Un po' - In larga misura - In misura molto larga | |
|  |  |  |  |

**PREVEDIBILITÀ**

|  | Sul suo posto di lavoro, è informato con largo anticipo riguardo ad esempio a decisioni importanti, cambiamenti o piani per il futuro? | - In misura molto ridotta - In piccola parte - Un po' - In larga misura - In misura molto larga | |
| --- | --- | --- | --- |
|  | Riceve tutte le informazioni di cui ha bisogno per svolgere bene il suo lavoro? | - In misura molto ridotta - In piccola parte - Un po' - In larga misura - In misura molto larga | |
|  |  |  |  |

**CONFLITTO DI RUOLO**

|  | Le vengono poste richieste contraddittorie al lavoro? | - In misura molto ridotta - In piccola parte - Un po' - In larga misura - In misura molto larga | |
| --- | --- | --- | --- |
|  | A volte deve fare cose che avrebbero dovuto essere fatte in un modo diverso? | - In misura molto ridotta - In piccola parte - Un po' - In larga misura - In misura molto larga | |
|  |  |  |  |

**SUPPORTO SOCIALE DA PARTE DEL SUPERVISORE**

|  | Con quale frequenza il suo diretto superiore è disposto ad ascoltare i suoi problemi sul lavoro, se necessario? | - Non ho un supervisore - Mai - Raramente - A volte - Spesso - Sempre | |
| --- | --- | --- | --- |
|  | Con quale frequenza riceve aiuto e supporto dal suo diretto superiore, se necessario? | - Non ho un supervisore - Mai - Raramente - A volte - Spesso - Sempre | |
|  |  |  |  |

**SUPPORTO SOCIALE DA PARTE DEI COLLEGHI**

|  | Con quale frequenza riceve aiuto e supporto dai suoi colleghi, se necessario? | - Non ho colleghi - Mai - Raramente - A volte - Spesso - Sempre | |
| --- | --- | --- | --- |
|  | Con quale frequenza i suoi colleghi sono disposti ad ascoltare i suoi problemi sul lavoro, se necessario? | - Non ho colleghi - Mai - Raramente - A volte - Spesso - Sempre | |
|  | Con quale frequenza i suoi colleghi le parlano di come svolge il suo lavoro? | - Non ho colleghi - Mai - Raramente - A volte - Spesso - Sempre | |
|  |  |  |  |

**SENSO DEL LAVORO**

|  | Il suo lavoro è significativo? | - In misura molto ridotta - In piccola parte - Un po' - In larga misura - In misura molto larga | |
| --- | --- | --- | --- |
|  | Sente che il lavoro che fa è importante? | - In misura molto ridotta - In piccola parte - Un po' - In larga misura - In misura molto larga | |
|  |  |  |  |

**IMPEGNO LAVORATIVO**

|  | Le seguenti affermazioni riguardano come si sente al lavoro. Se ha avuto questa sensazione, indichi quanto spesso l'ha provata. | | |
| --- | --- | --- | --- |
|  | Al lavoro, mi sento scoppiare di energia. | - Mai - Raramente - A volte - Spesso - Sempre | |
|  | Sono entusiasta del mio lavoro. | - Mai - Raramente - A volte - Spesso - Sempre | |
|  | Sono immerso nel mio lavoro | - Mai - Raramente - A volte - Spesso - Sempre | |
|  |  |  |  |

**CONFLITTO NELLA VITA LAVORATIVA**

|  | Ritiene che il suo lavoro prosciughi così tanto la sua energia da avere un effetto negativo sulla sua vita privata? | - In misura molto ridotta - In piccola parte - Un po' - In larga misura - In misura molto larga | |
| --- | --- | --- | --- |
|  | Ritiene che il suo lavoro richieda così tanto tempo da avere un effetto negativo sulla sua vita privata? | - In misura molto ridotta - In piccola parte - Un po' - In larga misura - In misura molto larga | |
|  |  |  |  |

**BURNOUT (MBI: Emotional Exhaustion+Depersonalization)**

Questions of this section are concealed for copyright reasons. See Schaufeli WB, Bakker AB, Hoogduin K, Schaap C, Kladler A. On the clinical validity of the Maslach Burnout Inventory and the Burnout Measure. Psychol Health. 2001;16(5):565–82.

Use questions 01. 02. 03. 06. 08. 13. 14. 16. 20 to assess Emotional Exhaustion and questions 05. 10. 11. 15. 22 to assess Depersonalization

**SATISFACTION WITH WORK – JOB SATISFACTION**

|  | Riguardo il suo lavoro in generale. Quanto è soddisfatto | | |
| --- | --- | --- | --- |
|  | Delle sue prospettive lavorative? | - Veramente insoddisfatto - Insoddisfatto - Né insoddisfatto né soddisfatto - Soddisfatto - Veramente soddisfatto | |
|  | Delle sue condizioni fisiche di lavoro? | - Veramente insoddisfatto - Insoddisfatto - Né insoddisfatto né soddisfatto - Soddisfatto - Veramente soddisfatto | |
|  | Del modo in cui vengono utilizzate le sue abilità? | - Veramente insoddisfatto - Insoddisfatto - Né insoddisfatto né soddisfatto - Soddisfatto - Veramente soddisfatto | |
|  | Del suo lavoro nel suo insieme, prendendo in considerazione tutto? | - Veramente insoddisfatto - Insoddisfatto - Né insoddisfatto né soddisfatto - Soddisfatto - Veramente soddisfatto | |
|  | Del suo stipendio? | - Veramente insoddisfatto - Insoddisfatto - Né insoddisfatto né soddisfatto - Soddisfatto - Veramente soddisfatto | |
|  |  |  |  |

**TURNOVER INTENTION**

|  | Ho intenzione di lasciare il mio attuale ospedale per un altro nel prossimo futuro | - Fortemente d’accordo - D’accordo - Né d’accordo né in disaccordo - In Disaccordo - Fortemente in disaccordo | |
| --- | --- | --- | --- |
|  | Ho intenzione di lasciare la mia professione sanitaria per un altro lavoro | - Fortemente d’accordo - D’accordo - Né d’accordo né in disaccordo - In Disaccordo - Fortemente in disaccordo | |
|  |  |  |  |

**SUGGESTION FOR IMPROVEMENT OF THE METEOR PROJECT**

|  | Dall'elenco sottostante, selezioni le tre cose principali che ritiene siano più efficaci nel ridurre il burnout e nel migliorare il benessere | - Limitare l'orario di lavoro dei medici - Fornire assistenza all'infanzia in loco - Migliorare il numero di personale medico - Migliorare il numero di personale infermieristico - Ridurre le chiamate notturne - Fornire formazione sulla resilienza - Creazione di tempo e luoghi per la meditazione e la riflessione - Adeguato rapporto personale/paziente - Ridurre gli straordinari - Consentire di trascorrere più tempo con i pazienti - Ridurre la burocrazia - Ridurre il carico di documentazione clinica (cartaceo/digitale) - Altro (scrivere; fornire una dichiarazione nella pagina successiva) | |
| --- | --- | --- | --- |
|  |  |  |  |

**Grazie per aver completato il questionario METEOR!**

Se vuole saperne di più sul nostro lavoro e sul progetto “METEOR”, visiti [www.meteorproject.eu](http://www.meteorproject.eu).

email: [survey@meteorproject.eu](mailto:survey@meteorproject.eu)

1. **Polish Questionnaire**

**3.1 Kwestionariusz dla lekarzy: aktualne doświadczenie zawodowe**

Szanowna Pani/Szanowny Panie

Zapraszamy Panią/Pana do wzięcia udziału w projekcie badawczym „Mental Health: Focus on Retention of Healthcare workers (METEOR)”. Badanie jest realizowane w oparciu o środki finansowe przyznawane przez Komisję Europejską w ramach Programu UE dla zdrowia (2014-2020) (Umowa nr 101018310). Głównym celem projektu METEOR jest wypracowanie działań mających na celu poprawę stanu zdrowia psychicznego oraz jakości życia lekarzy, a także innych pracowników ochrony zdrowia w krajach Unii Europejskiej.

Chcielibyśmy zapytać Panią/Pana o kwestie związane z postrzeganiem różnych obszarów środowiska pracy, takich jak m.in.: relacje z innymi pracownikami ochrony zdrowia, obciążenie pracą oraz ewentualny zamiar odejścia z zawodu. Wypełnienie ankiety sprawi, że będzie Pani/Pan mieć realny wpływ na poprawę sprawowanej opieki nad pacjentami we własnym środowisku pracy.

Zapewniamy, że badanie przeprowadzimy zgodnie z najwyższymi standardami naukowymi,

a Pani/Pana odpowiedzi są ściśle poufne. Ponadto pragniemy zapewnić, że w badaniu nie będą gromadzone dane personalne. Wypełnienie ankiety zajmie nie więcej niż 15-20 minut.

Z poważaniem,

Członkowie zespołu badawczego:

**O TOBIE**

|  | Płeć | - Mężczyzna - Kobieta - Inne |
| --- | --- | --- |
|  | Wiek | ………………. |
|  | Jaka jest Pani(a) sytuacja związana  z zamieszkaniem? | - Mieszkam samotnie - Mieszkam z partnerem/partnerką - Mieszkam z partnerem oraz dzieckiem/dziećmi - Mieszkam z przyjaciółmi/współlokatorami - Mieszkam z rodzicami - Inne |

**ZATRUDNIENIE**

|  | Szpital macierzysty | - UZ Leuven Academic hospital, Belgia - AZ Delta, Belgia - Uniwersyteckie Centrum Kliniczne, Polska - Szpital Wielospecjalistyczny w Jaworznie, Polska - Amsterdam University Medical Centre, Holandia - Spaarne Gasthuis, Holandia - Palermo University Hospital, Włochy - Local Health Facility of Agrigento, Włochy | |
| --- | --- | --- | --- |
|  | Forma zatrudnienia | - W pełnym wymiarze - W niepełnym wymiarze (< 100% etatu) | |
|  | Rodzaj umowy | - Na czas nieokreślony - Na czas określony | |
|  | Od ilu lat pracujesz w obecnym szpitalu? | - 1 rok lub mniej - 2 lata - … - 40 i więcej lat | |
|  | Czy pracujesz na nocne zmiany? | - Tak - Nie | |
|  | Specjalizacja | - Anestezjologia i intensywna terapia - Kardiologia - Dermatologia i wenerologia - Rehabilitaja medyczna - Gastroenterologia - Medycyna sądowa - Geriatria - Ginekologia i położnictwo - Chirurgia ogólna - Choroby wewnętrzne - Psychiatria dzieci i młodzieży - Psychiatria - Onkologia kliniczna - Neurochirurgia - Neurologia - Okulistyka - Medycyna nuklearna - Chirurgia urazowo-ortopedyczna - Otolaryngologia - Patomorfologia - Pediatria - Chirurgia plastyczna - Choroby płuc - Radioterapia onkologiczna - Reumatologia - Radiologia i diagnostyka obrazowa - Stomatologia, chirurgia szczękowo-twarzowa - Medycyna ratunkowa - Urologia - Medycyna pracy - Medycyna rodzinna - Inna…................................(proszę podać) | |
|  | Czy byłaś(eś) bezpośrednio zaangażowana(y) w pracę z pacjentami z potwierdzonym COVID-19? | - Tak - Jeśli Nie, proszę przejść do następnej sekcji | |
|  | Jak długo pracowałaś(eś) z pacjentami, u których potwierdzono COVID-19? | - 1 rok lub mniej - Więcej niż 1 rok | |
|  |  |  |  |

**WARUNKI PRACY**

|  | Szpital zapewnia wszystkie środki, które są mi potrzebne aby jak najlepiej wykonywać swoją pracę | - Zdecydowanie się zgadzam - Raczej się zgadzam - Ani się nie zgadzam, ani zgadzam - Raczej się nie zgadzam - Zdecydowanie się nie zgadzam | |
| --- | --- | --- | --- |
|  |  |  |  |

**PROBLEMY ZDROWOTNE PODCZAS OSTATNIEJ PRACY**

|  | Czy cierpiałaś(eś) na jakąkolwiek chorobę, niepełnosprawność lub inny problem fizyczny lub psychiczny spowodowany lub pogorszony przez pracę (poza wypadkami w pracy), w ciągu ostatnich trzech lat? | - Tak - Nie | |
| --- | --- | --- | --- |
|  |  |  |  |

**DOSTĘPNOŚĆ PERSONELU**

|  | Mamy wystarczającą liczbę pracowników w stosunku do obciążenia pracą | - Zdecydowanie się zgadzam - Raczej się zgadzam - Ani się nie zgadzam, ani zgadzam - Raczej się nie zgadzam - Zdecydowanie się nie zgadzam | |
| --- | --- | --- | --- |
|  | Pracownicy pracują po godzinach pracy tak długo, że może to być ze szkodą dla pacjenta | - Zdecydowanie się nie zgadzam - Raczej się nie zgadzam - Ani się nie zgadzam, ani zgadzam - Raczej się zgadzam - Zdecydowanie się zgadzam | |
|  | Zatrudnionych jest więcej pracowników tymczasowych niż jest to konieczne dla zapewnienia pacjentowi możliwie najlepszej opieki | - Zdecydowanie się nie zgadzam - Raczej się nie zgadzam - Ani się nie zgadzam, ani zgadzam - Raczej się zgadzam - Zdecydowanie się zgadzam | |
|  | Pracujemy w „trybie kryzysowym” – próbujemy wykonać zbyt wiele zadań i w zbyt krótkim czasie | - Zdecydowanie się nie zgadzam - Raczej się nie zgadzam - Ani się nie zgadzam, ani zgadzam - Raczej się zgadzam - Zdecydowanie się zgadzam | |
|  |  |  |  |

**TEMPO PRACY**

|  | Czy musisz pracować bardzo szybko? | - Nigdy - Rzadko - Czasami - Często - Zawsze | |
| --- | --- | --- | --- |
|  | Czy pracujesz w szybkim tempie przez cały dzień? | - W bardzo małym stopniu - W małym stopniu - Do pewnego stopnia - W dużym stopniu - W bardzo dużym stopniu | |
|  |  |  |  |

**WYMAGANIA ILOŚCIOWE**

|  | Czy obciążenie pracą jest rozłożone nierównomiernie – tak, że zadania gromadzą się? | - Nigdy - Rzadko - Czasami - Często - Zawsze | |
| --- | --- | --- | --- |
|  | Jak często brakuje Ci czasu na wykonanie wszystkich zadań? | - Nigdy - Rzadko - Czasami - Często - Zawsze | |
|  | Czy masz zaległości w pracy? | - Nigdy - Rzadko - Czasami - Często - Zawsze | |
|  | Czy masz wystarczająco dużo czasu na wykonywanie zadań w pracy? | - Zawsze - Często - Czasami - Rzadko - Nigdy | |
|  |  |  |  |

**WYMAGANIA POZNAWCZE**

|  | Czy podczas pracy musisz zwracać uwagę na wiele rzeczy? | - Nigdy - Rzadko - Czasami - Często - Zawsze | |
| --- | --- | --- | --- |
|  | Czy praca wymaga, abyś pamiętała/pamiętał o wielu rzeczach? | - Nigdy - Rzadko - Czasami - Często - Zawsze | |
|  | Czy praca wymaga od Ciebie umiejętności przedstawiania nowych pomysłów? | - Nigdy - Rzadko - Czasami - Często - Zawsze | |
|  | Czy praca wymaga podejmowania trudnych decyzji? | - Nigdy - Rzadko - Czasami - Często - Zawsze | |
|  |  |  |  |

**WYMAGANIA EMOCJONALNE**

|  | Czy praca stawia Cię w niepokojących emocjonalnie sytuacjach? | - Nigdy - Rzadko - Czasami - Często - Zawsze | |
| --- | --- | --- | --- |
|  | Czy w ramach swojej pracy musisz radzić sobie z problemami osobistymi innych ludzi? | - Nigdy - Rzadko - Czasami - Często - Zawsze | |
|  | Czy Twoja praca jest wymagająca emocjonalnie? | - W bardzo małym stopniu - W małym stopniu - Do pewnego stopnia - W dużym stopniu - W bardzo dużym stopniu | |
|  |  |  |  |

**KONTROLA W PRACY**

|  | Czy masz duży wpływ na decyzje dotyczące Twojej pracy? | - Nigdy - Rzadko - Czasami - Często - Zawsze | |
| --- | --- | --- | --- |
|  | Czy masz wpływ na ilość przydzielanej Ci pracy? | - Nigdy - Rzadko - Czasami - Często - Zawsze | |
|  | Czy masz wpływ na to, co robisz w pracy? | - Nigdy - Rzadko - Czasami - Często - Zawsze | |
|  | Czy masz wpływ na to JAK wykonujesz swoją pracę? | - Nigdy - Rzadko - Czasami - Często - Zawsze | |
|  |  |  |  |

**ZASTRASZANIE LUB ZNĘCANIE (*BULLYING*)**

|  | Czy kiedykolwiek w trakcie ostatnich 12 miesięcy doświadczyłaś(eś) w pracy zastraszania/znęcania? | - Tak, codziennie - Tak, każdego tygodnia - Tak, każdego miesiąca - Tak, kilka razy - Tak, tylko raz - Nie | |
| --- | --- | --- | --- |
|  |  |  |  |

**MOŻLIWOŚCI ROZWOJU**

|  | Czy masz możliwość nauczenia się czegoś nowego dzięki pracy? | - W bardzo małym stopniu - W małym stopniu - Do pewnego stopnia - W dużym stopniu - W bardzo dużym stopniu | |
| --- | --- | --- | --- |
|  | Czy masz możliwość wykorzystania swoich umiejętności i doświadczenia w pracy? | - W bardzo małym stopniu - W małym stopniu - Do pewnego stopnia - W dużym stopniu - W bardzo dużym stopniu | |
|  |  |  |  |

**PRZEWIDYWALNOŚĆ**

|  | Czy w swoim miejscu pracy jesteś informowana(y) z dużym yprzedzeniem o np. ważnych decyzjach, zmianach lub planach na przyszłość? | - W bardzo małym stopniu - W małym stopniu - Do pewnego stopnia - W dużym stopniu - W bardzo dużym stopniu | |
| --- | --- | --- | --- |
|  | Czy otrzymujesz wszystkie informacje, których potrzebujesz, aby dobrze ykonywać swoją pracę? | - W bardzo małym stopniu - W małym stopniu - Do pewnego stopnia - W dużym stopniu - W bardzo dużym stopniu | |
|  |  |  |  |

**KONFLIKT RÓL**

|  | Czy praca stawia Ci sprzeczne wymagania? | - W bardzo małym stopniu - W małym stopniu - Do pewnego stopnia - W dużym stopniu - W bardzo dużym stopniu | |
| --- | --- | --- | --- |
|  | Czy czasami musisz robić rzeczy, które należało zrobić inaczej? | - W bardzo małym stopniu - W małym stopniu - Do pewnego stopnia - W dużym stopniu - W bardzo dużym stopniu | |
|  |  |  |  |

**WSPARCIE SPOŁECZNE OD PRZEŁOŻONEGO**

|  | Jak często Twój bezpośredni przełożony jest gotów wysłuchać twoich problemów w pracy, jeśli to konieczne? | - Nie mam bezpośredniego przełożonego - Nigdy - Rzadko - Czasami - Często - Zawsze | |
| --- | --- | --- | --- |
|  | Jak często, w razie potrzeby, otrzymujesz pomoc i wsparcie od swojego bezpośredniego przełożonego? | - Nie mam bezpośredniego przełożonego - Nigdy - Rzadko - Czasami - Często - Zawsze | |
|  |  |  |  |

**WSPARCIE SPOŁECZNE OD KOLEGÓW**

|  | Jak często, w razie potrzeby, otrzymujesz pomoc i wsparcie od kolegów? | - Nie mam kolegów - Nigdy - Rzadko - Czasami - Często - Zawsze | |
| --- | --- | --- | --- |
|  | Jak często koledzy są gotowi wysłuchać Twoich problemów w pracy, jeśli zajdzie taka potrzeba? | - Nie mam kolegów - Nigdy - Rzadko - Czasami - Często - Zawsze | |
|  | Jak często Twoi koledzy rozmawiają z Tobą o tym, jak dobrze wykonujesz swoją pracę? | - Nie mam kolegów - Nigdy - Rzadko - Czasami - Często - Zawsze | |
|  |  |  |  |

**SENS PRACY**

|  | Czy Twoja praca ma sens? | - W bardzo małym stopniu - W małym stopniu - Do pewnego stopnia - W dużym stopniu - W bardzo dużym stopniu | |
| --- | --- | --- | --- |
|  | Czy odczuwasz, że Twoja praca jest ważna? | - W bardzo małym stopniu - W małym stopniu - Do pewnego stopnia - W dużym stopniu - W bardzo dużym stopniu | |
|  |  |  |  |

**ZAANGAŻOWANIE W PRACĘ**

|  | Poniższe stwierdzenia dotyczą Twojego samopoczucia w pracy. Jeśli miałaś(eś) to uczucie, wskaż, jak często je odczuwałaś(eś) | | |
| --- | --- | --- | --- |
|  | W pracy czuję się pełna(en) energii | - Nigdy - Rzadko - Czasami - Często - Zawsze | |
|  | Jestem entuzjastycznie nastawiona(y) do swojej pracy | - Nigdy - Rzadko - Czasami - Często - Zawsze | |
|  | Jestem pochłonięta(y) pracą | - Nigdy - Rzadko - Czasami - Często - Zawsze | |
|  |  |  |  |

**WYPALENIE ZAWODOWE**

|  | Czy czujesz, że praca wysysa z Ciebie tyle energii, że ma to negatywny wpływ na Twoje życie prywatne? | - W bardzo małym stopniu - W małym stopniu - Do pewnego stopnia - W dużym stopniu - W bardzo dużym stopniu | |
| --- | --- | --- | --- |
|  | Czy czujesz, że praca zabiera Ci tak dużo czasu, że wpływa to negatywnie na Twoje życie prywatne? | - W bardzo małym stopniu - W małym stopniu - Do pewnego stopnia - W dużym stopniu - W bardzo dużym stopniu | |
|  |  |  |  |

**BURNOUT (MBI: Emotional Exhaustion+Depersonalization)**

Questions of this section are concealed for copyright reasons. See Schaufeli WB, Bakker AB, Hoogduin K, Schaap C, Kladler A. On the clinical validity of the Maslach Burnout Inventory and the Burnout Measure. Psychol Health. 2001;16(5):565–82.

Use questions 01. 02. 03. 06. 08. 13. 14. 16. 20 to assess Emotional Exhaustion and questions 05. 10. 11. 15. 22 to assess Depersonalization

**SATYSFAKCJA Z PRACY**

|  | Ogólnie rzecz biorąc, w jakim stopniu jesteś zadowolony(a) z poszczególnych aspektów swojej pracy | | |
| --- | --- | --- | --- |
|  | Perspektywy rozwoju w pracy | - Bardzo niezadowolona(y) - Niezadowolona(y) - Ani niezadowolona(y), ani zadowolona(y) - Zadowolona(y) - Bardzo zadowolona(y) | |
|  | Fizyczne warunki pracy | - Bardzo niezadowolona(y) - Niezadowolona(y) - Ani niezadowolona(y), ani zadowolona(y) - Zadowolona(y) - Bardzo zadowolona(y) | |
|  | Sposób, w jaki wykorzystywane są moje umiejętności | - Bardzo niezadowolona(y) - Niezadowolona(y) - Ani niezadowolona(y), ani zadowolona(y) - Zadowolona(y) - Bardzo zadowolona(y) | |
|  | Praca jako całość, biorąc pod uwagę wszystkie jej elementy | - Bardzo niezadowolona(y) - Niezadowolona(y) - Ani niezadowolona(y), ani zadowolona(y) - Zadowolona(y) - Bardzo zadowolona(y) | |
|  | Wynagrodzenie | - Bardzo niezadowolona(y) - Niezadowolona(y) - Ani niezadowolona(y), ani zadowolona(y) - Zadowolona(y) - Bardzo zadowolona(y) | |
|  |  |  |  |

**ZAMIAR ZMIANY MIEJSCA PRACY**

|  | W najbliższym czasie mam zamiar zrezygnować z pracy w obecnym szpitalu  i zatrudnić się w innej placówce | - Zdecydowanie się zgadzam - Raczej się zgadzam - Ani się nie zgadzam, ani zgadzam - Raczej się nie zgadzam - Zdecydowanie się nie zgadzam | |
| --- | --- | --- | --- |
|  | Mam zamiar zrezygnować z pracy w sektorze ochrny zdrowia i zmienić zawód na inny | - Zdecydowanie się zgadzam - Raczej się zgadzam - Ani się nie zgadzam, ani zgadzam - Raczej się nie zgadzam - Zdecydowanie się nie zgadzam | |
|  |  |  |  |

**SUGESTIE ODNOŚNIE MOŻLIWOŚCI POPRAWY PROJEKTU METEOR**

|  | Z poniższej listy wybierz trzy najważniejsze działania, które Twoim zdaniem mogą najskuteczniej zmniejszyć wypalenie zawodowe i poprawić samopoczucie lekarzy sprawujących opiekę nad chorymi | - Ograniczenie liczby godzin pracy lekarzy - Zapewnienie opieki nad dziećmi personelu medycznego w miejscu pracy - Poprawa poziomu zatrudnienia pielęgniarek - Poprawa poziomu zatrudnienia lekarzy - Ograniczenie liczby nocnych wezwań - Zapewnienie szkolenia w zakresie odporności na stres w związku z wykonywaną pracą - Stworzenie możliwości w zakresie czasu i miejsca do medytacji i/lub refleksji - Adekwatny do potrzeb, stały wskaźnik zatrudnienia w szpitalu - Redukcja nadgodzin - Umożliwienie lekarzom spędzania większej ilości czasu z pacjentami - Redukcja biurokracji i obowiązujących procedur - Zmniejszenie obciążeń związanych z dokumentacją medyczną (papierową/cyfrową) - Inne (*proszę wpisać jakie*) | |
| --- | --- | --- | --- |
|  |  |  |  |

**Dziękujemy za wzięcie udziału w badaniu METEOR!**

Jeśli chce Pan/i dowiedzieć się więcej na temat naszej pracy oraz projektu „METEOR”, proszę odwiedzić stronę [www.meteorproject.eu](http://www.meteorproject.eu).

email: [survey@meteorproject.eu](mailto:survey@meteorproject.eu)

**3.2 Kwestionariusz dla pielęgniarek: aktualne doświadczenie zawodowe**

Szanowna Pani/Szanowny Panie

Zapraszamy Panią/Pana do wzięcia udziału w projekcie badawczym „Mental Health: Focus on Retention of Healthcare workers (METEOR)”. Badanie jest realizowane w oparciu o środki finansowe przyznawane przez Komisję Europejską w ramach Programu UE dla zdrowia (2014-2020) (Umowa nr 101018310). Głównym celem projektu METEOR jest wypracowanie działań mających na celu poprawę stanu zdrowia psychicznego oraz jakości życia pielęgniarek, a także innych pracowników ochrony zdrowia w krajach Unii Europejskiej.

Chcielibyśmy zapytać Panią/Pana o kwestie związane z postrzeganiem różnych obszarów środowiska pracy, takich jak m.in.: relacje z innymi pracownikami ochrony zdrowia, obciążenie pracą oraz ewentualny zamiar odejścia z zawodu. Wypełnienie ankiety sprawi, że będzie Pani/Pan mieć realny wpływ na poprawę sprawowanej opieki nad pacjentami we własnym środowisku pracy.

Zapewniamy, że badanie przeprowadzimy zgodnie z najwyższymi standardami naukowymi,

a Pani/Pana odpowiedzi są ściśle poufne. Ponadto pragniemy zapewnić, że w badaniu nie będą gromadzone dane personalne. Wypełnienie ankiety zajmie nie więcej niż 15-20 minut.

Z poważaniem,

Członkowie zespołu badawczego:

**O TOBIE**

|  | Płeć | - Mężczyzna - Kobieta - Inne |
| --- | --- | --- |
|  | Wiek | …………………….. |
|  | Jaka jest Pani(a) sytuacja związana  z zamieszkaniem? | - Mieszkam samotnie - Mieszkam z partnerem/partnerką - Mieszkam z partnerem oraz dzieckiem/dziećmi - Mieszkam z przyjaciółmi/współlokatorami - Mieszkam z rodzicami - Inne |
|  | Najwyższy osiągnięty poziom wykształcenia | - Wyższe licencjackie - Wyższe magisterskie - Doktorat |

**ZATRUDNIENIE**

|  | Szpital macierzysty | - UZ Leuven Academic hospital, Belgia - AZ Delta, Belgia - Uniwersyteckie Centrum Kliniczne, Polska - Szpital Wielospecjalistyczny w Jaworznie, Polska - Amsterdam University Medical Centre, Holandia - Spaarne Gasthuis, Holandia - Palermo University Hospital, Włochy - Local Health Facility of Agrigento, Włochy | |
| --- | --- | --- | --- |
|  | Forma zatrudnienia | - W pełnym wymiarze; - W niepełnym wymiarze (< 100% etatu) | |
|  | Rodzaj umowy | - Na czas nieokreślony - Na czas określony | |
|  | Od ilu lat pracujesz w obecnym szpitalu? | - 1 rok lub mniej - 2 lata - … - 40 i więcej lat | |
|  | Czy pracujesz na nocne zmiany? | - Tak - Nie | |
|  | Specjalizacja | - Anestezjologia i intensywna terapia - Kardiologia - Dermatologia i wenerologia - Rehabilitaja medyczna - Gastroenterologia - Medycyna sądowa - Geriatria - Ginekologia i położnictwo - Chirurgia ogólna - Choroby wewnętrzne - Psychiatria dzieci i młodzieży - Psychiatria - Onkologia kliniczna - Neurochirurgia - Neurologia - Okulistyka - Medycyna nuklearna - Chirurgia urazowo-ortopedyczna - Otolaryngologia - Patomorfologia - Pediatria - Chirurgia plastyczna - Choroby płuc - Radioterapia onkologiczna - Reumatologia - Radiologia i diagnostyka obrazowa - Stomatologia, chirurgia szczękowo-twarzowa - Medycyna ratunkowa - Urologia - Medycyna pracy - Medycyna rodzinna - Inna…................................(proszę podać) | |
|  | Czy byłaś(eś) bezpośrednio zaangażowana(y) w pracę z pacjentami z potwierdzonym COVID-19? | - Tak - Jeśli Nie, proszę przejść do następnej sekcji | |
|  | Jak długo pracowałaś(eś) z pacjentami, u których potwierdzono COVID-19? | - 1 rok lub mniej - Więcej niż 1 rok | |
|  |  |  |  |

**WARUNKI PRACY**

|  | Szpital zapewnia wszystkie środki, które są mi potrzebne aby jak najlepiej wykonywać swoją pracę | - Zdecydowanie się zgadzam - Raczej się zgadzam - Ani się nie zgadzam, ani zgadzam - Raczej się nie zgadzam - Zdecydowanie się nie zgadzam | |
| --- | --- | --- | --- |
|  |  |  |  |

**PROBLEMY ZDROWOTNE PODCZAS OSTATNIEJ PRACY**

|  | Czy cierpiałaś(eś) na jakąkolwiek chorobę, niepełnosprawność lub inny problem fizyczny lub psychiczny spowodowany lub pogorszony przez pracę (poza wypadkami w pracy), w ciągu ostatnich trzech lat? | - Tak - Nie | |
| --- | --- | --- | --- |
|  |  |  |  |

**DOSTĘPNOŚĆ PERSONELU**

|  | Mamy wystarczającą liczbę pracowników w stosunku do obciążenia pracą | - Zdecydowanie się zgadzam - Raczej się zgadzam - Ani się nie zgadzam, ani zgadzam - Raczej się nie zgadzam - Zdecydowanie się nie zgadzam | |
| --- | --- | --- | --- |
|  | Pracownicy pracują po godzinach pracy tak długo, że może to być ze szkodą dla pacjenta | - Zdecydowanie się nie zgadzam - Raczej się nie zgadzam - Ani się nie zgadzam, ani zgadzam - Raczej się zgadzam - Zdecydowanie się zgadzam | |
|  | Zatrudnionych jest więcej pracowników tymczasowych niż jest to konieczne dla zapewnienia pacjentowi możliwie najlepszej opieki | - Zdecydowanie się nie zgadzam - Raczej się nie zgadzam - Ani się nie zgadzam, ani zgadzam - Raczej się zgadzam - Zdecydowanie się zgadzam | |
|  | Pracujemy w „trybie kryzysowym” – próbujemy wykonać zbyt wiele zadań i w zbyt krótkim czasie | - Zdecydowanie się nie zgadzam - Raczej się nie zgadzam - Ani się nie zgadzam, ani zgadzam - Raczej się zgadzam - Zdecydowanie się zgadzam | |
|  |  |  |  |

**TEMPO PRACY**

|  | Czy musisz pracować bardzo szybko? | - Nigdy - Rzadko - Czasami - Często - Zawsze | |
| --- | --- | --- | --- |
|  | Czy pracujesz w szybkim tempie przez cały dzień? | - W bardzo małym stopniu - W małym stopniu - Do pewnego stopnia - W dużym stopniu - W bardzo dużym stopniu | |
|  |  |  |  |

**WYMAGANIA ILOŚCIOWE**

|  | Czy obciążenie pracą jest rozłożone nierównomiernie – tak, że zadania gromadzą się? | - Nigdy - Rzadko - Czasami - Często - Zawsze | |
| --- | --- | --- | --- |
|  | Jak często brakuje Ci czasu na wykonanie wszystkich zadań? | - Nigdy - Rzadko - Czasami - Często - Zawsze | |
|  | Czy masz zaległości w pracy? | - Nigdy - Rzadko - Czasami - Często - Zawsze | |
|  | Czy masz wystarczająco dużo czasu na wykonywanie zadań w pracy? | - Zawsze - Często - Czasami - Rzadko - Nigdy | |
|  |  |  |  |

**WYMAGANIA POZNAWCZE**

|  | Czy podczas pracy musisz zwracać uwagę na wiele rzeczy? | - Nigdy - Rzadko - Czasami - Często - Zawsze | |
| --- | --- | --- | --- |
|  | Czy praca wymaga, abyś pamiętała/pamiętał o wielu rzeczach? | - Nigdy - Rzadko - Czasami - Często - Zawsze | |
|  | Czy praca wymaga od Ciebie umiejętności przedstawiania nowych pomysłów? | - Nigdy - Rzadko - Czasami - Często - Zawsze | |
|  | Czy praca wymaga podejmowania trudnych decyzji? | - Nigdy - Rzadko - Czasami - Często - Zawsze | |
|  |  |  |  |

**WYMAGANIA EMOCJONALNE**

|  | Czy praca stawia Cię w niepokojących emocjonalnie sytuacjach? | - Nigdy - Rzadko - Czasami - Często - Zawsze | |
| --- | --- | --- | --- |
|  | Czy w ramach swojej pracy musisz radzić sobie z problemami osobistymi innych ludzi? | - Nigdy - Rzadko - Czasami - Często - Zawsze | |
|  | Czy Twoja praca jest wymagająca emocjonalnie? | - W bardzo małym stopniu - W małym stopniu - Do pewnego stopnia - W dużym stopniu - W bardzo dużym stopniu | |
|  |  |  |  |

**KONTROLA W PRACY**

|  | Czy masz duży wpływ na decyzje dotyczące Twojej pracy? | - Nigdy - Rzadko - Czasami - Często - Zawsze | |
| --- | --- | --- | --- |
|  | Czy masz wpływ na ilość przydzielanej Ci pracy? | - Nigdy - Rzadko - Czasami - Często - Zawsze | |
|  | Czy masz wpływ na to, co robisz w pracy? | - Nigdy - Rzadko - Czasami - Często - Zawsze | |
|  | Czy masz wpływ na to JAK wykonujesz swoją pracę? | - Nigdy - Rzadko - Czasami - Często - Zawsze | |
|  |  |  |  |

**ZASTRASZANIE LUB ZNĘCANIE (*BULLYING*)**

|  | Czy kiedykolwiek w trakcie ostatnich 12 miesięcy doświadczyłaś(eś) w pracy zastraszania/znęcania? | - Tak, codziennie - Tak, każdego tygodnia - Tak, każdego miesiąca - Tak, kilka razy - Tak, tylko raz - Nie | |
| --- | --- | --- | --- |
|  |  |  |  |

**MOŻLIWOŚCI ROZWOJU**

|  | Czy masz możliwość nauczenia się czegoś nowego dzięki pracy? | - W bardzo małym stopniu - W małym stopniu - Do pewnego stopnia - W dużym stopniu - W bardzo dużym stopniu | |
| --- | --- | --- | --- |
|  | Czy masz możliwość wykorzystania swoich umiejętności i doświadczenia w pracy? | - W bardzo małym stopniu - W małym stopniu - Do pewnego stopnia - W dużym stopniu - W bardzo dużym stopniu | |
|  |  |  |  |

**PRZEWIDYWALNOŚĆ**

|  | Czy w swoim miejscu pracy jesteś informowana(y) z dużym yprzedzeniem o np. ważnych decyzjach, zmianach lub planach na przyszłość? | - W bardzo małym stopniu - W małym stopniu - Do pewnego stopnia - W dużym stopniu - W bardzo dużym stopniu | |
| --- | --- | --- | --- |
|  | Czy otrzymujesz wszystkie informacje, których potrzebujesz, aby dobrze ykonywać swoją pracę? | - W bardzo małym stopniu - W małym stopniu - Do pewnego stopnia - W dużym stopniu - W bardzo dużym stopniu | |
|  |  |  |  |

**KONFLIKT RÓL**

|  | Czy praca stawia Ci sprzeczne wymagania? | - W bardzo małym stopniu - W małym stopniu - Do pewnego stopnia - W dużym stopniu - W bardzo dużym stopniu | |
| --- | --- | --- | --- |
|  | Czy czasami musisz robić rzeczy, które należało zrobić inaczej? | - W bardzo małym stopniu - W małym stopniu - Do pewnego stopnia - W dużym stopniu - W bardzo dużym stopniu | |
|  |  |  |  |

**WSPARCIE SPOŁECZNE OD PRZEŁOŻONEGO**

|  | Jak często Twój bezpośredni przełożony jest gotów wysłuchać twoich problemów w pracy, jeśli to konieczne? | - Nie mam bezpośredniego przełożonego - Nigdy - Rzadko - Czasami - Często - Zawsze | |
| --- | --- | --- | --- |
|  | Jak często, w razie potrzeby, otrzymujesz pomoc i wsparcie od swojego bezpośredniego przełożonego? | - Nie mam bezpośredniego przełożonego - Nigdy - Rzadko - Czasami - Często - Zawsze | |
|  |  |  |  |

**WSPARCIE SPOŁECZNE OD KOLEGÓW**

|  | Jak często, w razie potrzeby, otrzymujesz pomoc i wsparcie od kolegów? | - Nie mam kolegów - Nigdy - Rzadko - Czasami - Często - Zawsze | |
| --- | --- | --- | --- |
|  | Jak często koledzy są gotowi wysłuchać Twoich problemów w pracy, jeśli zajdzie taka potrzeba? | - Nie mam kolegów - Nigdy - Rzadko - Czasami - Często - Zawsze | |
|  | Jak często Twoi koledzy rozmawiają z Tobą o tym, jak dobrze wykonujesz swoją pracę? | - Nie mam kolegów - Nigdy - Rzadko - Czasami - Często - Zawsze | |
|  |  |  |  |

**SENS PRACY**

|  | Czy Twoja praca ma sens? | - W bardzo małym stopniu - W małym stopniu - Do pewnego stopnia - W dużym stopniu - W bardzo dużym stopniu | |
| --- | --- | --- | --- |
|  | Czy odczuwasz, że Twoja praca jest ważna? | - W bardzo małym stopniu - W małym stopniu - Do pewnego stopnia - W dużym stopniu - W bardzo dużym stopniu | |
|  |  |  |  |

**ZAANGAŻOWANIE W PRACĘ**

|  | Poniższe stwierdzenia dotyczą Twojego samopoczucia w pracy. Jeśli miałaś(eś) to uczucie, wskaż, jak często je odczuwałaś(eś) | | |
| --- | --- | --- | --- |
|  | W pracy czuję się pełna(en) energii | - Nigdy - Rzadko - Czasami - Często - Zawsze | |
|  | Jestem entuzjastycznie nastawiona(y) do swojej pracy | - Nigdy - Rzadko - Czasami - Często - Zawsze | |
|  | Jestem pochłonięta(y) pracą | - Nigdy - Rzadko - Czasami - Często - Zawsze | |
|  |  |  |  |

**WYPALENIE ZAWODOWE**

|  | Czy czujesz, że praca wysysa z Ciebie tyle energii, że ma to negatywny wpływ na Twoje życie prywatne? | - W bardzo małym stopniu - W małym stopniu - Do pewnego stopnia - W dużym stopniu - W bardzo dużym stopniu | |
| --- | --- | --- | --- |
|  | Czy czujesz, że praca zabiera Ci tak dużo czasu, że wpływa to negatywnie na Twoje życie prywatne? | - W bardzo małym stopniu - W małym stopniu - Do pewnego stopnia - W dużym stopniu - W bardzo dużym stopniu | |
|  |  |  |  |

**BURNOUT (MBI: Emotional Exhaustion+Depersonalization)**

Questions of this section are concealed for copyright reasons. See Schaufeli WB, Bakker AB, Hoogduin K, Schaap C, Kladler A. On the clinical validity of the Maslach Burnout Inventory and the Burnout Measure. Psychol Health. 2001;16(5):565–82.

Use questions 01. 02. 03. 06. 08. 13. 14. 16. 20 to assess Emotional Exhaustion and questions 05. 10. 11. 15. 22 to assess Depersonalization

**SATYSFAKCJA Z PRACY**

|  | Ogólnie rzecz biorąc, w jakim stopniu jesteś zadowolony(a) z poszczególnych aspektów swojej pracy | | |
| --- | --- | --- | --- |
|  | Perspektywy rozwoju w pracy | - Bardzo niezadowolona(y) - Niezadowolona(y) - Ani niezadowolona(y), ani zadowolona(y) - Zadowolona(y) - Bardzo zadowolona(y) | |
|  | Fizyczne warunki pracy | - Bardzo niezadowolona(y) - Niezadowolona(y) - Ani niezadowolona(y), ani zadowolona(y) - Zadowolona(y) - Bardzo zadowolona(y) | |
|  | Sposób, w jaki wykorzystywane są moje umiejętności | - Bardzo niezadowolona(y) - Niezadowolona(y) - Ani niezadowolona(y), ani zadowolona(y) - Zadowolona(y) - Bardzo zadowolona(y) | |
|  | Praca jako całość, biorąc pod uwagę wszystkie jej elementy | - Bardzo niezadowolona(y) - Niezadowolona(y) - Ani niezadowolona(y), ani zadowolona(y) - Zadowolona(y) - Bardzo zadowolona(y) | |
|  | Wynagrodzenie | - Bardzo niezadowolona(y) - Niezadowolona(y) - Ani niezadowolona(y), ani zadowolona(y) - Zadowolona(y) - Bardzo zadowolona(y) | |
|  |  |  |  |

**ZAMIAR ZMIANY MIEJSCA PRACY**

|  | W najbliższym czasie mam zamiar zrezygnować z pracy w obecnym szpitalu  i zatrudnić się w innej placówce | - Zdecydowanie się zgadzam - Raczej się zgadzam - Ani się nie zgadzam, ani zgadzam - Raczej się nie zgadzam - Zdecydowanie się nie zgadzam | |
| --- | --- | --- | --- |
|  | Mam zamiar zrezygnować z pracy w sektorze ochrny zdrowia i zmienić zawód na inny | - Zdecydowanie się zgadzam - Raczej się zgadzam - Ani się nie zgadzam, ani zgadzam - Raczej się nie zgadzam - Zdecydowanie się nie zgadzam | |
|  |  |  |  |

**SUGESTIE ODNOŚNIE MOŻLIWOŚCI POPRAWY PROJEKTU METEOR**

|  | Z poniższej listy wybierz trzy najważniejsze działania, które Twoim zdaniem mogą najskuteczniej zmniejszyć wypalenie zawodowe i poprawić samopoczucie lekarzy sprawujących opiekę nad chorymi | - Ograniczenie liczby godzin pracy lekarzy - Zapewnienie opieki nad dziećmi personelu medycznego w miejscu pracy - Poprawa poziomu zatrudnienia pielęgniarek - Poprawa poziomu zatrudnienia lekarzy - Ograniczenie liczby nocnych wezwań - Zapewnienie szkolenia w zakresie odporności na stres w związku z wykonywaną pracą - Stworzenie możliwości w zakresie czasu i miejsca do medytacji i/lub refleksji - Adekwatny do potrzeb, stały wskaźnik zatrudnienia w szpitalu - Redukcja nadgodzin - Umożliwienie lekarzom spędzania większej ilości czasu z pacjentami - Redukcja biurokracji i obowiązujących procedur - Zmniejszenie obciążeń związanych z dokumentacją medyczną (papierową/cyfrową) - Inne (*proszę wpisać jakie*) | |
| --- | --- | --- | --- |
|  |  |  |  |

**Dziękujemy za wzięcie udziału w badaniu METEOR!**

Jeśli chce Pan/i dowiedzieć się więcej na temat naszej pracy oraz projektu „METEOR”, proszę odwiedzić stronę [www.meteorproject.eu](http://www.meteorproject.eu).

email: [survey@meteorproject.eu](mailto:survey@meteorproject.eu)

1. **Dutch Questionnaire**

**4.1 Vragenlijst voor Artsen**

Beste,

Bedankt voor uw deelname aan onze studie over **job retentie, job tevredenheid en het welzijn bij artsen.** METEOR is een project dat wordt gefinancierd door de *European Union’s Health Programme 2014-2020* (overeenkomst 101018310) en dit project heeft als voornaamste doelstelling om zicht te krijgen op job retentie onder gezondheidswerkers, met een focus op de mentale gezondheid en job tevredenheid.

Wij willen u vragen een reeks vragen te beantwoorden over uw **werkervaringen,** inclusief uw perceptie over uw werkomgeving. De vragenlijst moet binnen drie weken na ontvangst van deze uitnodiging worden ingevuld en neemt ongeveer **10-15 minuten** in beslag. Als arts met kennis uit de eerste hand van uw ziekenhuis, zijn uw antwoorden van cruciaal belang en zullen ze worden gebruikt om de patiëntenzorg en uw werkomgeving te verbeteren.

Uw antwoorden op de vragen zijn **strikt persoonlijk en vertrouwelijk**. Elke vraag moet beantwoord worden. Gelieve de optie te kiezen die het best uw huidige gevoelens of meningen over het onderwerp weergeeft. Tijdens de periode van drie weken kunt u de vragenlijst op elk moment en wanneer het u uitkomt opnieuw raadplegen en verder invullen. U mag de vragenlijst slechts één keer invullen.

Contactgegevens van de Belgische onderzoekers:

**OVER JOU**

**Achtergrond**

|  | Wat is uw geslacht? | - Man - Vrouw - Andere |
| --- | --- | --- |
|  | Wat is uw leeftijd? | ………………….. |
|  | Hoe zou u uw huidige woonsituatie omschrijven? | - Alleenwonend - Smenwonend met partner - Samenwonend met partner en kind(eren), - Samenwonend met vrienden / huisgenoten - Wonend bij ouders - Andere |

**Werkcontext**

|  | In welk ziekenhuis werkt u momenteel? | - UZ Leuven Academisch ziekenhuis - AZ Delta - Spaarne Gasthuis - Amsterdam UMC | |
| --- | --- | --- | --- |
|  | Wat is uw huidig tewerkstellingsregime? | - Voltijds; - Deeltijds (alles < 100%) | |
|  | Onder welk type contract werkt u? | - Vast - Niet-vast (tijdelijk) | |
|  | Hoeveel jaar heeft u gewerkt als arts in dit ziekenhuis? | - Minder dan een jaar - Tussen een jaar en twee jaar - … - >40 jaar | |
|  | Werk je in nachtshiften? | - Jaa - Nee | |
|  | Wat is uw specialisatie? | - Anesthesie en reanimatie - Cardiologie - Dermatologie en venerologie - Fysische geneeskunde & revalidatie - Gastro-enterologie - Forensische geneeskunde - Geriatrie - Gynaecologie en verloskunde - Algemene chirurgie - Inwendige geneeskunde - Kinder- en jeugdpsychiatrie - Psychiatrie - Klinische biologie - Oncologie - Neurochirurgie - Neurologie - Oogheelkunde - Nucleaire geneeskunde - Orthopedische chirurgie - Otorinolaryngologie, Keel-Neus-Oorheelkunde (KNO) - Pathologische anatomie - Kindergeneeskunde - Plastische, reconstructieve en esthetische chirurgie - Pneumologie, longziekten - Radiotherapie - Reumatologie - Radiologie - Stomatologie, Mong-Kaak, en Aangezichts (MKA) - Spoedeisende geneeskunde, Urgentie - Urologie - Arbeidsgeneeskunde - Huisartsenpraktijk - Andere... (gelieve te specificeren) | |
|  | Was u vorig jaar rechtstreeks betrokken bij klinische activiteiten wat betreft de diagnose, behandeling en/of verpleging van patiënten met COVID-19? | - Ja - Nee, Bij nee, ga naar de sectie ‘werkomstandigheden’ | |
|  | Hoelang hebt u gewerkt aan de covid-19 frontlinie? | - Minder dan of gelijk aan één jaar - Meer dan een jaar | |
|  |  |  |  |

**Werkomstandigheden**

|  | Mijn ziekenhuis voorziet al het nodige materiaal zodat ik mijn job optimaal kan uitvoeren. | - Zeer mee eens - Mee eens - Noch mee eens, noch mee oneens - Mee oneens - Zeer mee oneens | |
| --- | --- | --- | --- |
|  |  |  |  |

**Gezondheidsproblemen Tijdens Uw Laatste Baan**

|  | Heeft u in uw laatste job - exclusief arbeidsongevallen - geleden aan een ziekte, arbeidsongeschiktheid of een ander lichamelijk of mentaal probleem dat door het werk werd veroorzaakt of verergerd? | - Ja - Nee | |
| --- | --- | --- | --- |
|  |  |  |  |

**PERSONEEL**

|  | We hebben voldoende personeel om de werklast aan te kunnen | - Sterk mee eens - Mee eens - Noch mee eens, noch mee oneens - Mee oneens - Sterk mee oneens | |
| --- | --- | --- | --- |
|  | Het personeel van deze eenheid werkt meer dan goed is voor de patiëntenzorg. | - Sterk mee oneens - Mee oneens - Noch mee eens, noch mee oneens - Mee eens - Sterk mee eens | |
|  | We gebruiken meer tijdelijk personeel dan goed is voor de patiëntenzorg. | - Sterk mee oneens - Mee oneens - Noch mee eens, noch mee oneens - Mee eens - Sterk mee eens | |
|  | We werken vaak in 'crisismodus' en proberen te veel, te snel te doen. | - Sterk mee oneens - Mee oneens - Noch mee eens, noch mee oneens - Mee eens - Sterk mee eens | |
|  |  |  |  |

**Werktempo**

|  | Moet u zeer snel werken? | - Nooit - Zelden - Soms - Vaak - Altijd | |
| --- | --- | --- | --- |
|  | Werkt u aan een hoog tempo gedurende de hele dag? | - In zeer kleine mate - In kleine mate - Enigszins - In belangrijke mate - In zeer belangrijke mate | |
|  |  |  |  |

**Kwantitatieve werkeisen**

|  | Is uw werkbelasting onregelmatig verdeeld, zodat het werk zich opstapelt? | - Nooit - Zelden - Soms - Vaak - Altijd | |
| --- | --- | --- | --- |
|  | Hoe vaak heeft u niet genoeg tijd om al uw taken op het werk af te werken? | - Nooit - Zelden - Soms - Vaak - Altijd | |
|  | Hoe vaak geraakt u achterop met uw werk? | - Nooit - Zelden - Soms - Vaak - Altijd | |
|  | Hoe vaak heeft u voldoende tijd om uw werktaken uit te voeren? | - Nooit - Zelden - Soms - Vaak - Altijd | |
|  |  |  |  |

**Cognitieve werkeisen**

|  | Moet u tijdens uw werk veel dingen in het oog houden? | - Nooit - Zelden - Soms - Vaak - Altijd | |
| --- | --- | --- | --- |
|  | Vereist uw werk van u dat u veel zaken moet  onthoudenn? | - Nooit - Zelden - Soms - Vaak - Altijd | |
|  | Vereist uw werk dat u goed bent in het aanbrengen van nieuwe ideeën? | - Nooit - Zelden - Soms - Vaak - Altijd | |
|  | Vereist uw werk dat u moeilijke beslissingen moet nemen? | - Nooit - Zelden - Soms - Vaak - Altijd | |
|  |  |  |  |

**Emotionele werkeisen**

|  | Wordt u door uw werk in emotioneel moeilijke situaties gedwongen? | - Nooit - Zelden - Soms - Vaak - Altijd | |
| --- | --- | --- | --- |
|  | Houdt uw werk in dat u te maken krijgt met de persoonlijke problemen van andere mensen? | - Nooit - Zelden - Soms - Vaak - Altijd | |
|  | Is uw werk emotioneel veeleisend? | - In zeer kleine mate - In kleine mate - Enigszins - In belangrijke mate - In zeer belangrijke mate | |
|  |  |  |  |

**INFLUENCE AT WORK**

|  | Heeft u een grote mate van invloed op beslissingen betreffende uw werk? | - Nooit - Zelden - Soms - Vaak - Altijd | |
| --- | --- | --- | --- |
|  | Kunt u de hoeveelheid werk die u wordt toegewezen beïnvloeden? | - Nooit - Zelden - Soms - Vaak - Altijd | |
|  | Heeft u enige invloed op wat u op uw werk doet? | - Nooit - Zelden - Soms - Vaak - Altijd | |
|  | Heeft u enige invloed op hoe u uw werk uitvoert? | - Nooit - Zelden - Soms - Vaak - Altijd | |
|  |  |  |  |

**Pestgedrag**

|  | Bent u blootgesteld aan pestgedrag op de werkvloer gedurende de laatste 12 maanden? | - Ja, dagelijks - Ja, wekelijks - Ja, maandelijks - Ja, enkele keren - Ja, één keer - Nee | |
| --- | --- | --- | --- |
|  |  |  |  |

**Ontwikkelingsmogelijkheden**

|  | Heeft u de mogelijkheid om via uw werk nieuwe dingen te leren? | - In zeer kleine mate - In kleine mate - Enigszins - In belangrijke mate - In zeer belangrijke mate | |
| --- | --- | --- | --- |
|  | Kunt u uw vaardigheden of expertise in uw werk gebruiken? | - In zeer kleine mate - In kleine mate - Enigszins - In belangrijke mate - In zeer belangrijke mate | |
|  |  |  |  |

**Voorspelbaarheid**

|  | Wordt u op uw werkplek ruimschoots van te voren geïnformeerd omtrent bijvoorbeeld belangrijke beslissingen, veranderingen of toekomstplannen? | - In zeer kleine mate - In kleine mate - Enigszins - In belangrijke mate - In zeer belangrijke mate | |
| --- | --- | --- | --- |
|  | Ontvangt u alle nodige informatie om uw werk goed te kunnen uitvoeren? | - In zeer kleine mate - In kleine mate - Enigszins - In belangrijke mate - In zeer belangrijke mate | |
|  |  |  |  |

**Rolconflict**

|  | Worden er u tijdens het werk tegenstrijdige eisen opgelegd? | - In zeer kleine mate - In kleine mate - Enigszins - In belangrijke mate - In zeer belangrijke mate | |
| --- | --- | --- | --- |
|  | Moet u soms dingen doen die op een andere manier hadden moeten uitgevoerd worden? | - In zeer kleine mate - In kleine mate - Enigszins - In belangrijke mate - In zeer belangrijke mate | |
|  |  |  |  |

**Sociale ondersteuning van leidinggevende**

|  | Als u het nodig zou hebben, hoe dikwijls zou uw onmiddellijke leidinggevende bereid zijn om te luisteren naar uw problemen op het werk? | - Ik heb geen leidinggevende - Nooit - Zelden - Soms - Vaak - Altijd | |
| --- | --- | --- | --- |
|  | Als u het nodig zou hebben, hoe dikwijls zou u hulp en ondersteuning krijgen van uw onmiddellijke leidinggevende? | - Ik heb geen leidinggevende - Nooit - Zelden - Soms - Vaak - Altijd | |
|  |  |  |  |

**Sociale Steun van collega’s**

|  | Als u het nodig zou hebben, hoe dikwijls zou u hulp en ondersteuning krijgen van uw collega’s? | - Ik heb geen collega’s - Nooit - Zelden - Soms - Vaak - Altijd | |
| --- | --- | --- | --- |
|  | Als u het nodig zou hebben, hoe dikwijls zouden uw collega’s bereid zijn om te luisteren naar uw problemen op het werk? | - Ik heb geen collega’s - Nooit - Zelden - Soms - Vaak - Altijd | |
|  | Hoe dikwijls hebben uw collega’s het met u over hoe goed u uw werk uitvoert? | - Ik heb geen collega’s - Nooit - Zelden - Soms - Vaak - Altijd | |
|  |  |  |  |

**Zinvolheid van het werk**

|  | Is uw werk zinvol? | - In zeer kleine mate - In kleine mate - Enigszins - In belangrijke mate - In zeer belangrijke mate | |
| --- | --- | --- | --- |
|  | Heeft u het gevoel dat het werk dat u doet belangrijk is? | - In zeer kleine mate - In kleine mate - Enigszins - In belangrijke mate - In zeer belangrijke mate | |
|  |  |  |  |

**BEVLOGENHEID**

|  | De volgende 3 stellingen gaan over hoe u u zich op uw werk voelt. Als u dit gevoel heeft gehad, geef dan aan hoe vaak u dit voelde. | | |
| --- | --- | --- | --- |
|  | Op mijn werk bruis ik van energie | - Nooit - Zelden - Soms - Vaak - Altijd | |
|  | Ik ben enthousiast over mijn werk | - Nooit - Zelden - Soms - Vaak - Altijd | |
|  | Ik ga helemaal op in mijn werk | - Nooit - Zelden - Soms - Vaak - Altijd | |
|  |  |  |  |

**WERK-PRIVE CONFLICT**

|  | Heeft u het gevoel dat uw werk zoveel van uw energie vergt, dat dit een negatief effect heeft op uw privéleven? | - In zeer kleine mate - In kleine mate - Enigszins - In belangrijke mate - In zeer belangrijke mate | |
| --- | --- | --- | --- |
|  | Heeft u het gevoel dat uw werk zoveel van uw tijd vergt, dat dit een negatief effect heeft op uw privéleven? | - In zeer kleine mate - In kleine mate - Enigszins - In belangrijke mate - In zeer belangrijke mate | |
|  |  |  |  |

**BURNOUT (MBI: Emotional Exhaustion+Depersonalization)**

Questions of this section are concealed for copyright reasons. See Schaufeli WB, Bakker AB, Hoogduin K, Schaap C, Kladler A. On the clinical validity of the Maslach Burnout Inventory and the Burnout Measure. Psychol Health. 2001;16(5):565–82.

Use questions 01. 02. 03. 06. 08. 13. 14. 16. 20 to assess Emotional Exhaustion and questions 05. 10. 11. 15. 22 to assess Depersonalization

**JOBTEVREDENHEID**

|  | Over uw werk in het algemeen. Hoe tevreden bent u over … | | |
| --- | --- | --- | --- |
|  | uw werkvooruitzichten? | - Zeer ontevreden - Ontevreden - Neutraal - Tevreden - Zeer tevreden | |
|  | de fysieke werkomstandigheden? | - Zeer ontevreden - Ontevreden - Neutraal - Tevreden - Zeer tevreden | |
|  | de manier waarop uw mogelijkheden worden gebruikt? | - Zeer ontevreden - Ontevreden - Neutraal - Tevreden - Zeer tevreden | |
|  | uw job in zijn geheel, alles in beschouwing genomen? | - Zeer ontevreden - Ontevreden - Neutraal - Tevreden - Zeer tevreden | |
|  | uw loon? | - Zeer ontevreden - Ontevreden - Neutraal - Tevreden - Zeer tevreden | |
|  |  |  |  |

**VERLOOP INTENTIE**

|  | Ik ben van plan om mijn huidig ziekenhuis te verlaten voor een ander ziekenhuis in de nabije toekomst. | - Zeer mee eens , - Mee eens - Noch mee eens, noch mee oneens - Mee oneens - Zeer mee oneens | |
| --- | --- | --- | --- |
|  | Ik ben van plan om mijn zorgberoep te verlaten voor een andere job | - Zeer mee eens , - Mee eens - Noch mee eens, noch mee oneens - Mee oneens - Zeer mee oneens | |
|  |  |  |  |

**SUGGESTIES VOOR VERBETERING**

|  | Kies uit onderstaande lijst de drie dingen die volgens u het meest effectief zouden zijn om het welzijn te verbeteren | - Beperking van de werkuren van artsen - Zorg voor kinderopvang ter plaatse - Verbetering van het personeelsbestand van de verpleegkundigen (meer aanwervingen,…) - Verbetering van het personeelsbestand van de artsen (meer aanwervingen,…) - Vermindering van nachtelijke oproeptaken - Weerbaarheidstraining geven - Zorg voor tijd en plaatsen om te mediteren en te bezinnen - Adequate verhouding vast personeel - Minder overwerk - Artsen meer tijd aan patiënten laten besteden - Vermindering van bureaucratie en administratieve rompslomp - Vermindering van de last van klinische documentatie (op papier/digitaal) - Andere (gelieve hier te noteren; verklaring op de volgende bladzijde opnemen) | |
| --- | --- | --- | --- |
|  |  |  |  |

**Dank u voor het invullen van de METEOR-vragenlijst!**

Als u meer wilt weten over ons werk en het 'METEOR'-project, verwijzen we u graag door naar onze website: [www.meteorproject.eu](http://www.meteorproject.eu)!

**4.2 Vragenlijst voor Verpleegkundigen**

Beste,

Bedankt voor uw deelname aan onze studie over **job retentie, job tevredenheid en het welzijn bij verpleegkundigen.** METEOR is een project dat wordt gefinancierd door de *European Union’s Health Programme 2014-2020* (overeenkomst 101018310) en dit project heeft als voornaamste doelstelling om zicht te krijgen op job retentie onder gezondheidswerkers, met een focus op de mentale gezondheid en job tevredenheid.

Wij willen u vragen een reeks vragen te beantwoorden over uw **werkervaringen,** inclusief uw perceptie over uw werkomgeving. De vragenlijst moet binnen drie weken na ontvangst van deze uitnodiging worden ingevuld en neemt ongeveer **10-15 minuten** in beslag. Als verpleegkundige met kennis uit de eerste hand van uw ziekenhuis, zijn uw antwoorden van cruciaal belang en zullen ze worden gebruikt om de patiëntenzorg en uw werkomgeving te verbeteren.

Uw antwoorden op de vragen zijn **strikt persoonlijk en vertrouwelijk**. Elke vraag moet beantwoord worden. Gelieve de optie te kiezen die het best uw huidige gevoelens of meningen over het onderwerp weergeeft. Tijdens de periode van drie weken kunt u de vragenlijst op elk moment en wanneer het u uitkomt opnieuw raadplegen en verder invullen. U mag de vragenlijst slechts één keer invullen.

**OVER JOU**

**Achtergrond**

|  | Wat is uw geslacht? | - Man - Vrouw - Andere |
| --- | --- | --- |
|  | Wat is uw leeftijd? | …………………….. |
|  | Hoe zou u uw huidige woonsituatie omschrijven? | - Alleenwonend - Smenwonend met partner - Samenwonend met partner en kind(eren), - Samenwonend met vrienden / huisgenoten - Wonend bij ouders - Andere |
|  | Wat is uw hoogst behaalde opleidingsniveau? | - Bachelorsdiploma - Masterdiploma - Doctoraat |

**Werkcontext**

|  | In welk ziekenhuis werkt u momenteel? | - UZ Leuven Academisch ziekenhuis - AZ Delta - Spaarne Gasthuis - Amsterdam UMC | |
| --- | --- | --- | --- |
|  | Wat is uw huidig tewerkstellingsregime? | - Voltijds; - Deeltijds (alles < 100%) | |
|  | Onder welk type contract werkt u? | - Vast - Niet-vast (tijdelijk) | |
|  | Hoeveel jaar heeft u gewerkt als arts in dit ziekenhuis? | - Minder dan een jaar - Tussen een jaar en twee jaar - … - >40 jaar | |
|  | Werk je in nachtshiften? | - Jaa - Nee | |
|  | Wat is uw specialisatie? | - Anesthesie en reanimatie - Cardiologie - Dermatologie en venerologie - Fysische geneeskunde & revalidatie - Gastro-enterologie - Forensische geneeskunde - Geriatrie - Gynaecologie en verloskunde - Algemene chirurgie - Inwendige geneeskunde - Kinder- en jeugdpsychiatrie - Psychiatrie - Klinische biologie - Oncologie - Neurochirurgie - Neurologie - Oogheelkunde - Nucleaire geneeskunde - Orthopedische chirurgie - Otorinolaryngologie, Keel-Neus-Oorheelkunde (KNO) - Pathologische anatomie - Kindergeneeskunde - Plastische, reconstructieve en esthetische chirurgie - Pneumologie, longziekten - Radiotherapie - Reumatologie - Radiologie - Stomatologie, Mong-Kaak, en Aangezichts (MKA) - Spoedeisende geneeskunde, Urgentie - Urologie - Arbeidsgeneeskunde - Huisartsenpraktijk - Andere... (gelieve te specificeren) | |
|  | Was u vorig jaar rechtstreeks betrokken bij klinische activiteiten wat betreft de diagnose, behandeling en/of verpleging van patiënten met COVID-19? | - Ja - Nee, Bij nee, ga naar de sectie ‘werkomstandigheden’ | |
|  | Hoelang hebt u gewerkt aan de covid-19 frontlinie? | - Minder dan of gelijk aan één jaar - Meer dan een jaar | |
|  |  |  |  |

**Werkomstandigheden**

|  | Mijn ziekenhuis voorziet al het nodige materiaal zodat ik mijn job optimaal kan uitvoeren. | - Zeer mee eens - Mee eens - Noch mee eens, noch mee oneens - Mee oneens - Zeer mee oneens | |
| --- | --- | --- | --- |
|  |  |  |  |

**Gezondheidsproblemen Tijdens Uw Laatste Baan**

|  | Heeft u in uw laatste job - exclusief arbeidsongevallen - geleden aan een ziekte, arbeidsongeschiktheid of een ander lichamelijk of mentaal probleem dat door het werk werd veroorzaakt of verergerd? | - Ja - Nee | |
| --- | --- | --- | --- |
|  |  |  |  |

**PERSONEEL**

|  | We hebben voldoende personeel om de werklast aan te kunnen | - Sterk mee eens - Mee eens - Noch mee eens, noch mee oneens - Mee oneens - Sterk mee oneens | |
| --- | --- | --- | --- |
|  | Het personeel van deze eenheid werkt meer dan goed is voor de patiëntenzorg. | - Sterk mee oneens - Mee oneens - Noch mee eens, noch mee oneens - Mee eens - Sterk mee eens | |
|  | We gebruiken meer tijdelijk personeel dan goed is voor de patiëntenzorg. | - Sterk mee oneens - Mee oneens - Noch mee eens, noch mee oneens - Mee eens - Sterk mee eens | |
|  | We werken vaak in 'crisismodus' en proberen te veel, te snel te doen. | - Sterk mee oneens - Mee oneens - Noch mee eens, noch mee oneens - Mee eens - Sterk mee eens | |
|  |  |  |  |

**Werktempo**

|  | Moet u zeer snel werken? | - Nooit - Zelden - Soms - Vaak - Altijd | |
| --- | --- | --- | --- |
|  | Werkt u aan een hoog tempo gedurende de hele dag? | - In zeer kleine mate - In kleine mate - Enigszins - In belangrijke mate - In zeer belangrijke mate | |
|  |  |  |  |

**Kwantitatieve werkeisen**

|  | Is uw werkbelasting onregelmatig verdeeld, zodat het werk zich opstapelt? | - Nooit - Zelden - Soms - Vaak - Altijd | |
| --- | --- | --- | --- |
|  | Hoe vaak heeft u niet genoeg tijd om al uw taken op het werk af te werken? | - Nooit - Zelden - Soms - Vaak - Altijd | |
|  | Hoe vaak geraakt u achterop met uw werk? | - Nooit - Zelden - Soms - Vaak - Altijd | |
|  | Hoe vaak heeft u voldoende tijd om uw werktaken uit te voeren? | - Nooit - Zelden - Soms - Vaak - Altijd | |
|  |  |  |  |

**Cognitieve werkeisen**

|  | Moet u tijdens uw werk veel dingen in het oog houden? | - Nooit - Zelden - Soms - Vaak - Altijd | |
| --- | --- | --- | --- |
|  | Vereist uw werk van u dat u veel zaken moet  onthoudenn? | - Nooit - Zelden - Soms - Vaak - Altijd | |
|  | Vereist uw werk dat u goed bent in het aanbrengen van nieuwe ideeën? | - Nooit - Zelden - Soms - Vaak - Altijd | |
|  | Vereist uw werk dat u moeilijke beslissingen moet nemen? | - Nooit - Zelden - Soms - Vaak - Altijd | |
|  |  |  |  |

**Emotionele werkeisen**

|  | Wordt u door uw werk in emotioneel moeilijke situaties gedwongen? | - Nooit - Zelden - Soms - Vaak - Altijd | |
| --- | --- | --- | --- |
|  | Houdt uw werk in dat u te maken krijgt met de persoonlijke problemen van andere mensen? | - Nooit - Zelden - Soms - Vaak - Altijd | |
|  | Is uw werk emotioneel veeleisend? | - In zeer kleine mate - In kleine mate - Enigszins - In belangrijke mate - In zeer belangrijke mate | |
|  |  |  |  |

**INFLUENCE AT WORK**

|  | Heeft u een grote mate van invloed op beslissingen betreffende uw werk? | - Nooit - Zelden - Soms - Vaak - Altijd | |
| --- | --- | --- | --- |
|  | Kunt u de hoeveelheid werk die u wordt toegewezen beïnvloeden? | - Nooit - Zelden - Soms - Vaak - Altijd | |
|  | Heeft u enige invloed op wat u op uw werk doet? | - Nooit - Zelden - Soms - Vaak - Altijd | |
|  | Heeft u enige invloed op hoe u uw werk uitvoert? | - Nooit - Zelden - Soms - Vaak - Altijd | |
|  |  |  |  |

**Pestgedrag**

|  | Bent u blootgesteld aan pestgedrag op de werkvloer gedurende de laatste 12 maanden? | - Ja, dagelijks - Ja, wekelijks - Ja, maandelijks - Ja, enkele keren - Ja, één keer - Nee | |
| --- | --- | --- | --- |
|  |  |  |  |

**Ontwikkelingsmogelijkheden**

|  | Heeft u de mogelijkheid om via uw werk nieuwe dingen te leren? | - In zeer kleine mate - In kleine mate - Enigszins - In belangrijke mate - In zeer belangrijke mate | |
| --- | --- | --- | --- |
|  | Kunt u uw vaardigheden of expertise in uw werk gebruiken? | - In zeer kleine mate - In kleine mate - Enigszins - In belangrijke mate - In zeer belangrijke mate | |
|  |  |  |  |

**Voorspelbaarheid**

|  | Wordt u op uw werkplek ruimschoots van te voren geïnformeerd omtrent bijvoorbeeld belangrijke beslissingen, veranderingen of toekomstplannen? | - In zeer kleine mate - In kleine mate - Enigszins - In belangrijke mate - In zeer belangrijke mate | |
| --- | --- | --- | --- |
|  | Ontvangt u alle nodige informatie om uw werk goed te kunnen uitvoeren? | - In zeer kleine mate - In kleine mate - Enigszins - In belangrijke mate - In zeer belangrijke mate | |
|  |  |  |  |

**Rolconflict**

|  | Worden er u tijdens het werk tegenstrijdige eisen opgelegd? | - In zeer kleine mate - In kleine mate - Enigszins - In belangrijke mate - In zeer belangrijke mate | |
| --- | --- | --- | --- |
|  | Moet u soms dingen doen die op een andere manier hadden moeten uitgevoerd worden? | - In zeer kleine mate - In kleine mate - Enigszins - In belangrijke mate - In zeer belangrijke mate | |
|  |  |  |  |

**Sociale ondersteuning van leidinggevende**

|  | Als u het nodig zou hebben, hoe dikwijls zou uw onmiddellijke leidinggevende bereid zijn om te luisteren naar uw problemen op het werk? | - Ik heb geen leidinggevende - Nooit - Zelden - Soms - Vaak - Altijd | |
| --- | --- | --- | --- |
|  | Als u het nodig zou hebben, hoe dikwijls zou u hulp en ondersteuning krijgen van uw onmiddellijke leidinggevende? | - Ik heb geen leidinggevende - Nooit - Zelden - Soms - Vaak - Altijd | |
|  |  |  |  |

**Sociale Steun van collega’s**

|  | Als u het nodig zou hebben, hoe dikwijls zou u hulp en ondersteuning krijgen van uw collega’s? | - Ik heb geen collega’s - Nooit - Zelden - Soms - Vaak - Altijd | |
| --- | --- | --- | --- |
|  | Als u het nodig zou hebben, hoe dikwijls zouden uw collega’s bereid zijn om te luisteren naar uw problemen op het werk? | - Ik heb geen collega’s - Nooit - Zelden - Soms - Vaak - Altijd | |
|  | Hoe dikwijls hebben uw collega’s het met u over hoe goed u uw werk uitvoert? | - Ik heb geen collega’s - Nooit - Zelden - Soms - Vaak - Altijd | |
|  |  |  |  |

**Zinvolheid van het werk**

|  | Is uw werk zinvol? | - In zeer kleine mate - In kleine mate - Enigszins - In belangrijke mate - In zeer belangrijke mate | |
| --- | --- | --- | --- |
|  | Heeft u het gevoel dat het werk dat u doet belangrijk is? | - In zeer kleine mate - In kleine mate - Enigszins - In belangrijke mate - In zeer belangrijke mate | |
|  |  |  |  |

**BEVLOGENHEID**

|  | De volgende 3 stellingen gaan over hoe u u zich op uw werk voelt. Als u dit gevoel heeft gehad, geef dan aan hoe vaak u dit voelde. | | |
| --- | --- | --- | --- |
|  | Op mijn werk bruis ik van energie | - Nooit - Zelden - Soms - Vaak - Altijd | |
|  | Ik ben enthousiast over mijn werk | - Nooit - Zelden - Soms - Vaak - Altijd | |
|  | Ik ga helemaal op in mijn werk | - Nooit - Zelden - Soms - Vaak - Altijd | |
|  |  |  |  |

**WERK-PRIVE CONFLICT**

|  | Heeft u het gevoel dat uw werk zoveel van uw energie vergt, dat dit een negatief effect heeft op uw privéleven? | - In zeer kleine mate - In kleine mate - Enigszins - In belangrijke mate - In zeer belangrijke mate | |
| --- | --- | --- | --- |
|  | Heeft u het gevoel dat uw werk zoveel van uw tijd vergt, dat dit een negatief effect heeft op uw privéleven? | - In zeer kleine mate - In kleine mate - Enigszins - In belangrijke mate - In zeer belangrijke mate | |
|  |  |  |  |

**BURNOUT (MBI: Emotional Exhaustion+Depersonalization)**

Questions of this section are concealed for copyright reasons. See Schaufeli WB, Bakker AB, Hoogduin K, Schaap C, Kladler A. On the clinical validity of the Maslach Burnout Inventory and the Burnout Measure. Psychol Health. 2001;16(5):565–82.

Use questions 01. 02. 03. 06. 08. 13. 14. 16. 20 to assess Emotional Exhaustion and questions 05. 10. 11. 15. 22 to assess Depersonalization

**JOBTEVREDENHEID**

|  | Over uw werk in het algemeen. Hoe tevreden bent u over … | | |
| --- | --- | --- | --- |
|  | uw werkvooruitzichten? | - Zeer ontevreden - Ontevreden - Neutraal - Tevreden - Zeer tevreden | |
|  | de fysieke werkomstandigheden? | - Zeer ontevreden - Ontevreden - Neutraal - Tevreden - Zeer tevreden | |
|  | de manier waarop uw mogelijkheden worden gebruikt? | - Zeer ontevreden - Ontevreden - Neutraal - Tevreden - Zeer tevreden | |
|  | uw job in zijn geheel, alles in beschouwing genomen? | - Zeer ontevreden - Ontevreden - Neutraal - Tevreden - Zeer tevreden | |
|  | uw loon? | - Zeer ontevreden - Ontevreden - Neutraal - Tevreden - Zeer tevreden | |
|  |  |  |  |

**VERLOOP INTENTIE**

|  | Ik ben van plan om mijn huidig ziekenhuis te verlaten voor een ander ziekenhuis in de nabije toekomst. | - Zeer mee eens , - Mee eens - Noch mee eens, noch mee oneens - Mee oneens - Zeer mee oneens | |
| --- | --- | --- | --- |
|  | Ik ben van plan om mijn zorgberoep te verlaten voor een andere job | - Zeer mee eens , - Mee eens - Noch mee eens, noch mee oneens - Mee oneens - Zeer mee oneens | |
|  |  |  |  |

**SUGGESTIES VOOR VERBETERING**

|  | Kies uit onderstaande lijst de drie dingen die volgens u het meest effectief zouden zijn om het welzijn te verbeteren | - Beperking van de werkuren van artsen - Zorg voor kinderopvang ter plaatse - Verbetering van het personeelsbestand van de verpleegkundigen (meer aanwervingen,…) - Verbetering van het personeelsbestand van de artsen (meer aanwervingen,…) - Vermindering van nachtelijke oproeptaken - Weerbaarheidstraining geven - Zorg voor tijd en plaatsen om te mediteren en te bezinnen - Adequate verhouding vast personeel - Minder overwerk - Artsen meer tijd aan patiënten laten besteden - Vermindering van bureaucratie en administratieve rompslomp - Vermindering van de last van klinische documentatie (op papier/digitaal) - Andere (gelieve hier te noteren; verklaring op de volgende bladzijde opnemen) | |
| --- | --- | --- | --- |
|  |  |  |  |

**Dank u voor het invullen van de METEOR-vragenlijst!**

Als u meer wilt weten over ons werk en het 'METEOR'-project, verwijzen we u graag door naar onze website: [www.meteorproject.eu](http://www.meteorproject.eu)!


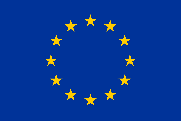


This project was funded by the European Union’s Health Programme (2014 - 2020).

*The content of this document represents the views of the authors only and is their sole responsibility; it cannot be considered to reflect the views of the European Commission and/or the* *European Health and Digital Executive Agency (HaDEA) or any other body of the European Union. The European Commission and the Agency do not accept any responsibility for use that may be made of the information it contains.*

***Proprietary rights***

*This document contains information which is proprietary to the METEOR consortium. Neither this document nor the information contained herein shall be used, duplicated or communicated by any means to any third party, in whole or parts, except with the prior written consent of the METEOR consortium.*

1. **Table S1 Sample distribution by sex and age of 381 physicians by country of the Meteor Survey**

|  | Overall  (n=381) | Belgium  (n=158) | Italy  (n=105) | Netherlands  (n=112) | Poland  (n=6) |
| --- | --- | --- | --- | --- | --- |
| Sex |  |  |  |  |  |
| Female | 229 (60.1 %) | 96 (60.8 %) | 52 (49.5 %) | 78 (69.6 %) | 3 (50.0 %) |
| Male | 152 (39.9 %) | 62 (39.2 %) | 53 (50.5 %) | 34 (30.4 %) | 3 (50.0 %) |
| Age (M±SD) | 44.7 (10.2) | 44.7 (10.2) | 44.7 (10.2) | 44.7 (10.2) | 44.7 (10.2) |

1. **Table S2 Indices of overall fit of alternative SEM model of the MTI**^§^ **for physicians**

| Model^§§§^ | Structure^§^ | Chi-square^§§^ | $df$ | CFI | TLI | SRMR | CD | RMSEA (p-value) |
| --- | --- | --- | --- | --- | --- | --- | --- | --- |
| One-Factor | all items | 8383 | 1769 | 0.450 | 0.431 | 0.105 | 0.96 | 0.099 (<0.001) |
| Two-factors | Esogenous: (jd*,jr*,we*,js*,ee*,dp*)  Endogenous: IL | 8346 | 1768 | 0.453 | 0.434 | 0.105 | 0.96 | 0.099 (<0.001) |
| Three- factors | Esogenous: (jd*,ee*,dp*),(jr*,js*,we*)  Endogenous: IL | 7391 | 1766 | 0.532 | 0.515 | 0.096 | 1.00 | 0.091 (<0.001) |
| Seven- factors | Esogenous: JD, JR, WE, JS, EE, DP  Endogenous: IL | 7057 | 1763 | 0.560 | 0.543 | 0.198 | 1.00 | 0.089 (<0.001) |
| Seven- factors1 | Esogenous: JD, JR  Endogenous: WE, JS, EE, DP, IL  Covariances: (EE,DP),(JD,JR) | 6182 | 1755 | 0.632 | 0.616 | 0.091 | 0.99 | 0.081 (<0.001) |
| Five-factors | Esogenous: JD, JR  Endogenous: JS, DP, IL  Covariances: (EE,DP),(JD,JR) | 4262 | 1745 | 0.791 | 0.781 | 0.084 | 0.99 | 0.062 (<0.001) |
| MTI for physicians | Esogenous: JD, JR  Endogenous: JS, DP, IL Covariances: (EE,DP),(JD,JR), (EE,JS) plus other significant covariances with measurement variables | 3183 | 1557 | 0.856 | 0.847 | 0.076 | 0.99 | 0.052 (0.068) |

^§^abbreviations: MTI (*T*urnover *I*ntention according to *M*eteor), JD (Job demands), JR (Job resources), WE (work engagement), JS (job satisfaction), EE (emotional exhaustion), DP (depersonalization) and IL (intention to leave) , jd* (measurables for JD), jr* (measurables for JR), we* (measurables for WE), js* (measurables for JS), ee* (measurables for EE), dp* (measurables for DP).

^§§§^Seven models are: the one-factor model joining together all items of the questionnaire, the two-factors model (IL as endogenous and all other items joined together as exogenous), the three-factors model (IL as endogenous; the items of JD, EE and DP joined together and the items of JR, WE and JS joined together, these latter as two exogenous), the seven-factors model (IL as endogenous; JD, JR, WE, JS, EE and DP as six exogenous); the seven-factors1 model (MTI model augmented of all significant covariance paths between pairs of latent variables); the five-factors model (seven-factors1 minus WE and EE); the MTI for physicians (five-factors augmented of all significant covariance paths between latent variables and between latent and observable variables).

1. **Table S3 Results of Confirmatory factor analysis:** **path coefficients of the measurement component of the MTI^§^ model for physicians**

| Latent costruct | Domain | Measurables | Path Coefficient | 95% CI | | P>z |
| --- | --- | --- | --- | --- | --- | --- |
| JD | staffing | jd_staff1 | 0.53 | 0.45 | 0.61 | <0.001 |
|  |  | jd_staff2 | 0.27 | 0.17 | 0.37 | <0.001 |
|  |  | jd_staff3 | -0.28 | -0.38 | -0.18 | <0.001 |
|  |  | jd_staff4 | 0.61 | 0.54 | 0.68 | <0.001 |
|  | work pace | jd_wp1 | 0.64 | 0.57 | 0.71 | <0.001 |
|  |  | jd_wp2 | 0.57 | 0.49 | 0.64 | <0.001 |
|  | quantitative demands | jd_dem1 | 0.69 | 0.63 | 0.75 | <0.001 |
|  |  | jd_dem2 | 0.68 | 0.61 | 0.74 | <0.001 |
|  |  | jd_dem3 | 0.58 | 0.51 | 0.66 | <0.001 |
|  |  | jd_dem4 | 0.68 | 0.62 | 0.74 | <0.001 |
|  | cognitive demands | jd_cogn1 | 0.49 | 0.4 | 0.57 | <0.001 |
|  |  | jd_cogn2 | 0.39 | 0.30 | 0.48 | <0.001 |
|  |  | jd_cogn3 | 0.15 | 0.05 | 0.26 | <0.001 |
|  |  | jd_cogn4 | 0.39 | 0.30 | 0.48 | <0.001 |
|  | emotional demands | jd_emo1 | 0.53 | 0.45 | 0.61 | <0.001 |
|  |  | jd_emo2 | 0.35 | 0.26 | 0.45 | <0.001 |
|  |  | jd_emo3 | 0.37 | 0.27 | 0.46 | <0.001 |
|  | role conflicts | jd_confl1 | 0.53 | 0.45 | 0.61 | <0.001 |
|  |  | jd_confl2 | 0.47 | 0.38 | 0.55 | <0.001 |
|  | work-life conflicts | jd_life1 | 0.64 | 0.57 | 0.70 | <0.001 |
|  |  | jd_life2 | 0.69 | 0.63 | 0.75 | <0.001 |
|  | bullying | jd_bul | 0.17 | 0.07 | 0.28 | <0.001 |
| JR | influence at work | jr_infl1 | 0.34 | 0.24 | 0.44 | <0.001 |
|  |  | jr_infl2 | 0.27 | 0.17 | 0.38 | <0.001 |
|  |  | jr_infl3 | 0.33 | 0.23 | 0.43 | <0.001 |
|  |  | jr_infl4 | 0.39 | 0.30 | 0.49 | <0.001 |
|  | possibility of development | jr_dev1 | 0.59 | 0.51 | 0.67 | <0.001 |
|  |  | jr_dev2 | 0.59 | 0.51 | 0.66 | <0.001 |
|  | predicatibility | jr_pred1 | 0.62 | 0.55 | 0.70 | <0.001 |
|  |  | jr_pred2 | 0.65 | 0.58 | 0.72 | <0.001 |
|  | social support from the supervisor | jr_superv1 | 0.56 | 0.49 | 0.64 | <0.001 |
|  |  | jr_superv2 | 0.60 | 0.53 | 0.68 | <0.001 |
|  | social support from collegues | jr_collegue1 | 0.54 | 0.46 | 0.62 | <0.001 |
|  |  | jr_collegue2 | 0.59 | 0.51 | 0.66 | <0.001 |
|  |  | jr_collegue3 | 0.32 | 0.22 | 0.42 | <0.001 |
|  | meaning of work | jr_mean1 | 0.42 | 0.33 | 0.51 | <0.001 |
|  |  | jr_mean2 | 0.34 | 0.24 | 0.43 | <0.001 |
| JS* | | sati1 | 0.80 | 0.76 | 0.84 | <0.001 |
|  |  | sati2 | 0.71 | 0.65 | 0.76 | <0.001 |
|  |  | sati3 | 0.79 | 0.75 | 0.84 | <0.001 |
|  |  | sati4 | 0.86 | 0.82 | 0.89 | <0.001 |
|  |  | sati5 | 0.45 | 0.37 | 0.54 | <0.001 |
| EE* | | ee1 | 0.83 | 0.78 | 0.87 | <0.001 |
|  |  | ee2 | 0.83 | 0.78 | 0.86 | <0.001 |
|  |  | ee3 | 0.74 | 0.69 | 0.79 | <0.001 |
|  |  | ee20 | 0.83 | 0.78 | 0.86 | <0.001 |
|  |  | ee8 | 0.83 | 0.78 | 0.87 | <0.001 |
|  |  | ee13 | 0.82 | 0.75 | 0.89 | <0.001 |
|  |  | ee14 | 0.63 | 0.56 | 0.70 | <0.001 |
|  |  | ee6 | 0.50 | 0.42 | 0.57 | <0.001 |
|  |  | ee16 | 0.46 | 0.38 | 0.54 | <0.001 |
| DP* | | dp5 | 0.58 | 0.50 | 0.66 | <0.001 |
|  |  | dp10 | 0.88 | 0.83 | 0.93 | <0.001 |
|  |  | dp11 | 1.09 | 0.93 | 1.24 | <0.001 |
|  |  | dp15 | 0.46 | 0.38 | 0.55 | <0.001 |
|  |  | dp22 | 0.33 | 0.23 | 0.43 | <0.001 |
| IL* | | il1 | 0.69 | 0.60 | 0.79 | <0.001 |
|  |  | il2 | 0.61 | 0.52 | 0.71 | <0.001 |

*Only one domain for this latent construct

^§^abbreviations: MTI (*T*urnover *I*ntention according to *M*eteor), JD (Job demands), JR (Job resources), WE (work engagement), JS (job satisfaction), EE (emotional exhaustion), DP (depersonalization) and IL (intention to leave) , jd* (measurables for JD), jr* (measurables for JR), we* (measurables for WE), js* (measurables for JS), ee* (measurables for EE), dp* (measurables for DP).

1. **Table S4 Sample distribution by sex and age of 1350 nurses by country of the Meteor Survey**

|  | Overall  (n=1,350) | Belgium  (n=857) | Italy  (n=86) | Netherlands  (n=344) | Poland  (n=63) |
| --- | --- | --- | --- | --- | --- |
| Sex |  |  |  |  |  |
| Female | 1,119 (83 %) | 716 (84 %) | 51 (59 %) | 291 (85 %) | 61 (97 %) |
| Male | 231 (17 %) | 141 (16 %) | 35 (41 %) | 53 (15 %) | 2 (3.2 %) |
| Age (M±SD) | 43.8 (11.8 %) | 43.7 (10.9 %) | 45.8 (11.7 %) | 42.6 (14.0 %) | 50.3 (8.8 %) |

1. **Table S5 Indices of overall fit of alternative SEM model of the MTI^§^ for nurses**

| Model | Structure | Chi-square^§^ | d.f. | CFI | TLI | SRMR | CD | RMSEA (p-value) |
| --- | --- | --- | --- | --- | --- | --- | --- | --- |
| One-Factor | all items | 22678 | 1769 | 0.465 | 0.447 | 0.098 | 0.96 | 0.094 (p<0.001) |
| Two-factors | Esogenous: (jd*,jr*,we*,js*,ee*,dp*)  Endogenous: IL | 22353 | 1768 | 0.473 | 0.455 | 0.097 | 0.96 | 0.093 (p<0.001) |
| Three- factors | Esogenous: (jd*,ee*,dp*),(jr*,js*,we*)  Endogenous: IL | 19464 | 1766 | 0.547 | 0.531 | 0.090 | 0.99 | 0.086 (p<0.001) |
| Seven- factors | Esogenous: JD, JR, WE, JS, EE, DP  Endogenous: IL | 17903 | 1763 | 0.587 | 0.571 | 0.186 | 1.00 | 0.082 (p<0.001) |
| Seven factors1 | Esogenous: JD, JR  Endogenous: WE, JS, EE, DP, IL Covariances: (EE,DP),(JD,JR) | 15176 | 1756 | 0.657 | 0.642 | 0.082 | 0.99 | 0.075 (p<0.001) |
| Five-factors | Esogenous: JD, JR  Endogenous: JS, DP, IL  Covariances: (EE,DP),(JD,JR), (JS,WE) | 15079 | 1756 | 0.659 | 0.645 | 0.082 | 0.99 | 0.075 (p<0.001) |
| MTI for nurses | Esogenous: JD, JR  Endogenous: JS, DP, IL  Covariances: (EE,DP),(JD,JR), (JS,WE), plus other significant covariances with measurement variables | 6376 | 1723 | 0.881 | 0.874 | 0.069 | 0.97 | 0.045 (p=1.000) |

^§^abbreviations: MTI (*T*urnover *I*ntention according to *M*eteor), JD (Job demands), JR (Job resources), WE (work engagement), JS (job satisfaction), EE (emotional exhaustion), DP (depersonalization) and IL (intention to leave) , jd* (measurables for JD), jr* (measurables for JR), we* (measurables for WE), js* (measurables for JS), ee* (measurables for EE), dp* (measurables for DP).

^§§§^Seven models are: the one-factor model joining together all items of the questionnaire, the two-factors model (IL as endogenous and all other items joined together as exogenous), the three-factors model (IL as endogenous; the items of JD, EE and DP joined together and the items of JR, WE and JS joined together, these latter as two exogenous), the seven-factors model (IL as endogenous; JD, JR, WE, JS, EE and DP as six exogenous); the seven-factors1 model (MTI model augmented of all significant covariance paths between pairs of latent variables); the five-factors model (seven-factors1 minus WE and EE); the MTI for nurses (five-factors augmented of all significant covariance paths between latent variables and between latent and observable variables).

1. **Table S6 Results of confirmatory factor analysis: path coefficients of the measurement component of the MTI^§^ model for nurses**

| Latent costruct | Domain | Measurables | Path Coefficient | 95% CI | | P>z |
| --- | --- | --- | --- | --- | --- | --- |
| JD | staffing | jd_staff1 | 0.60 | 0.56 | 0.64 | <0.001 |
|  |  | jd_staff2 | 0.46 | 0.42 | 0.51 | <0.001 |
|  |  | jd_staff3 | -0.14 | -0.2 | -0.09 | <0.001 |
|  |  | jd_staff4 | 0.64 | 0.6 | 0.67 | <0.001 |
|  | work pace | jd_wp1 | 0.63 | 0.59 | 0.66 | <0.001 |
|  |  | jd_wp2 | 0.58 | 0.54 | 0.62 | <0.001 |
|  | quantitative demands | jd_dem1 | 0.60 | 0.57 | 0.64 | <0.001 |
|  |  | jd_dem2 | 0.65 | 0.62 | 0.69 | <0.001 |
|  |  | jd_dem3 | 0.62 | 0.58 | 0.66 | <0.001 |
|  |  | jd_dem4 | 0.59 | 0.56 | 0.63 | <0.001 |
|  | cognitive demands | jd_cogn1 | 0.34 | 0.29 | 0.39 | <0.001 |
|  |  | jd_cogn2 | 0.32 | 0.27 | 0.38 | <0.001 |
|  |  | jd_cogn3 | 0.13 | 0.07 | 0.18 | <0.001 |
|  |  | jd_cogn4 | 0.35 | 0.29 | 0.4 | <0.001 |
|  | emotional demands | jd_emo1 | 0.47 | 0.43 | 0.52 | <0.001 |
|  |  | jd_emo2 | 0.26 | 0.21 | 0.32 | <0.001 |
|  |  | jd_emo3 | 0.44 | 0.39 | 0.49 | <0.001 |
|  | role conflicts | jd_confl1 | 0.48 | 0.43 | 0.53 | <0.001 |
|  |  | jd_confl2 | 0.47 | 0.42 | 0.51 | <0.001 |
|  | work-life conflicts | jd_life1 | 0.62 | 0.58 | 0.66 | <0.001 |
|  |  | jd_life2 | 0.59 | 0.55 | 0.63 | <0.001 |
|  | bullying | jd_bul | 0.21 | 0.15 | 0.26 | <0.001 |
| JR | influence at work | jr_infl1 | 0.25 | 0.2 | 0.31 | <0.001 |
|  |  | jr_infl2 | 0.26 | 0.21 | 0.32 | <0.001 |
|  |  | jr_infl3 | 0.35 | 0.29 | 0.4 | <0.001 |
|  |  | jr_infl4 | 0.34 | 0.28 | 0.39 | <0.001 |
|  | possibility of development | jr_dev1 | 0.52 | 0.48 | 0.57 | <0.001 |
|  |  | jr_dev2 | 0.51 | 0.46 | 0.55 | <0.001 |
|  | predicatibility | jr_pred1 | 0.54 | 0.49 | 0.58 | <0.001 |
|  |  | jr_pred2 | 0.58 | 0.54 | 0.63 | <0.001 |
|  | social support from the supervisor | jr_superv1 | 0.59 | 0.55 | 0.64 | <0.001 |
|  |  | jr_superv2 | 0.59 | 0.55 | 0.64 | <0.001 |
|  | social support from collegues | jr_collegue1 | 0.37 | 0.31 | 0.42 | <0.001 |
|  |  | jr_collegue2 | 0.37 | 0.32 | 0.42 | <0.001 |
|  |  | jr_collegue3 | 0.24 | 0.18 | 0.3 | <0.001 |
|  | meaning of work | jr_mean1 | 0.46 | 0.42 | 0.51 | <0.001 |
|  |  | jr_mean2 | 0.47 | 0.42 | 0.52 | <0.001 |
| WE* | | work_enga1 | 0.72 | 0.68 | 0.75 | <0.001 |
|  |  | work_enga2 | 0.85 | 0.82 | 0.88 | <0.001 |
|  |  | work_enga3 | 0.66 | 0.62 | 0.69 | <0.001 |
| JS* | | sati1 | 0.77 | 0.75 | 0.8 | <0.001 |
|  |  | sati2 | 0.65 | 0.62 | 0.69 | <0.001 |
|  |  | sati3 | 0.70 | 0.66 | 0.73 | <0.001 |
|  |  | sati4 | 0.80 | 0.77 | 0.82 | <0.001 |
|  |  | sati5 | 0.40 | 0.36 | 0.45 | <0.001 |
| EE* | | ee1 | 0.81 | 0.79 | 0.83 | <0.001 |
|  |  | ee2 | 0.77 | 0.74 | 0.79 | <0.001 |
|  |  | ee3 | 0.75 | 0.73 | 0.78 | <0.001 |
|  |  | ee20 | 0.82 | 0.8 | 0.84 | <0.001 |
|  |  | ee8 | 0.82 | 0.8 | 0.84 | <0.001 |
|  |  | ee13 | 0.73 | 0.7 | 0.75 | <0.001 |
|  |  | ee14 | 0.63 | 0.6 | 0.67 | <0.001 |
|  |  | ee6 | 0.56 | 0.52 | 0.6 | <0.001 |
|  |  | ee16 | 0.52 | 0.48 | 0.56 | <0.001 |
| DP* | | dp5 | 0.57 | 0.53 | 0.61 | <0.001 |
|  |  | dp10 | 0.83 | 0.81 | 0.86 | <0.001 |
|  |  | dp11 | 0.84 | 0.82 | 0.87 | <0.001 |
|  |  | dp15 | 0.56 | 0.52 | 0.6 | <0.001 |
|  |  | dp22 | 0.42 | 0.37 | 0.46 | <0.001 |
| IL* | | il1 | 0.70 | 0.66 | 0.74 | <0.001 |
|  |  | il2 | 0.81 | 0.77 | 0.85 | <0.001 |

*Only one domain for this latent construct

^§^abbreviations: MTI (*T*urnover *I*ntention according to *M*eteor), JD (Job demands), JR (Job resources), WE (work engagement), JS (job satisfaction), EE (emotional exhaustion), DP (depersonalization) and IL (intention to leave) , jd* (measurables for JD), jr* (measurables for JR), we* (measurables for WE), js* (measurables for JS), ee* (measurables for EE), dp* (measurables for DP).
